# Optimizing Efficient RNAi-Mediated Control of Hemipteran Pests (Psyllids, Leafhoppers, Whitefly): Modified Pyrimidines in dsRNA Triggers

**DOI:** 10.3390/plants10091782

**Published:** 2021-08-26

**Authors:** Wayne Brian Hunter, William M. Wintermantel

**Affiliations:** 1 U.S. Horticultural Research Laboratory, U.S. Department of Agriculture, Agriculture Research Service, Subtropical Insects Res., Fort Pierce, FL 34945, USA; 2 U.S. Department of Agriculture, Agriculture Research Service, Crop Improvement and Protection Research, Salinas, CA 93905, USA; bill.wintermantel@usda.gov

**Keywords:** cassava, citrus, dsRNA, Hemiptera, huanglongbing, leafhopper, RNAi, psyllid, Solanaceous, whitefly

## Abstract

The advantages from exogenously applied RNAi biopesticides have yet to be realized in through commercialization due to inconsistent activity of the dsRNA trigger, and the activity level of RNAi suppression. This has prompted research on improving delivery methods for applying exogenous dsRNA into plants and insects for the management of pests and pathogens. Another aspect to improve RNAi activity is the incorporation of modified 2′-F pyrimidine nucleotides into the dsRNA trigger. Modified dsRNA incorporating 32–55% of the 2′-F- nucleotides produced improved RNAi activity that increased insect mortality by 12–35% greater than non-modified dsRNA triggers of the same sequence. These results were repeatable across multiple Hemiptera: the Asian citrus psyllid (*Diaphorina citri,* Liviidae); whitefly (*Bemisia tabaci,* Aleyroididae); and the glassy-winged sharpshooter (*Homalodisca vitripennis*, Cicadellidae). Studies using siRNA with modified 2′-F- pyrimidines in mammalian cells show they improved resistance to degradation from nucleases, plus result in greater RNAi activity, due to increase concentrations and improved binding affinity to the mRNA target. Successful RNAi biopesticides of the future will be able to increase RNAi repeatability in the field, by incorporating modifications of the dsRNA, such as 2′-F- pyrimidines, that will improve delivery after applied to fruit trees or crop plants, with increased activity after ingestion by insects. Costs of RNA modification have decreased significantly over the past few years such that biopesticides can now compete on pricing with commercial chemical products.

## 1. Introduction

**Figure 1:**
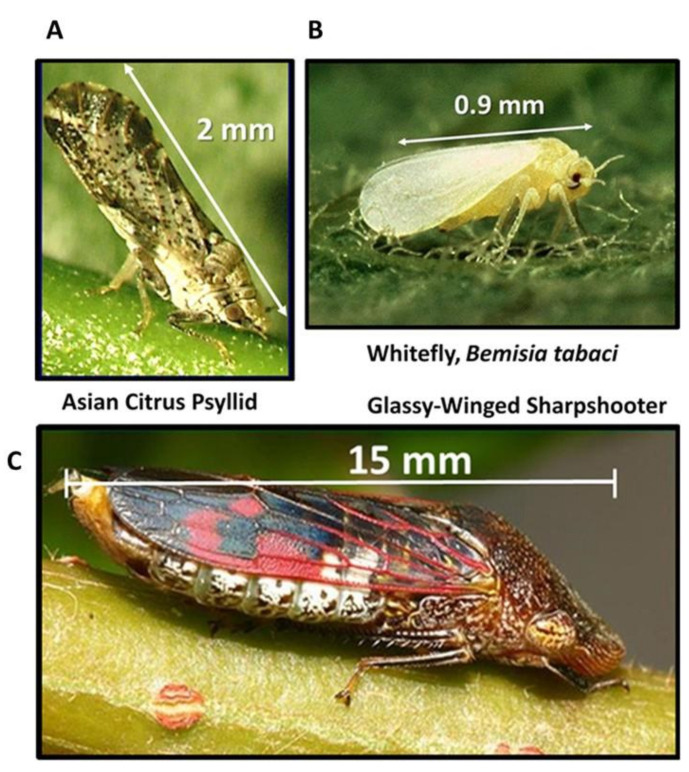
Hemipteran Insect Vectors of Crop Pandemics. (**A**) Asian citrus psyllid, *Diaphorina citri* Kuwayama, 1908 (Liviidae) (https://www.cabi.org/isc/datasheet/18615, accessed 15 July 2021); (**B**) Silverleaf Whitefly, *Bemisia tabaci* (Gennadius, 1889) MEAMI, (Aleyroididae) (https://www.cabi.org/isc/datasheet/8925, accessed 15 July 2021); (**C**) Glassy-winged Sharpshooter Leafhopper, *Homalodisca vitripennis* (Germar, 1821) (Cicadellidae) (https://www.cabi.org/isc/datasheet/27561#todescription, accessed 15 July 2021) (Figure S1. Global Distribution Maps, Supplemental Material).

This study demonstrates the increased activity of dsRNA’s using modified pyrimidines to effectively manage hemipteran vectors and pests in citrus trees, olive trees, grapevines, cassava, vegetables and other agricultural crops. Effective RNAi biopesticides need improvements in activity and resistance to degradation by nucleases [1,2,3] to be suitable to incorporate into area wide of insect vectors and the plant pathogens they transmit. RNA-targeted strategies focus on the most serious insect vectors as viable approaches to reduce the spread of bacterial and viral pathogens causing global crop pandemics [4,5,6,7]. The tree insect vectors evaluated in this study includes: the Asian citrus psyllid, *Diaphorina citri* Kuwayama 1908, (Figure 1A), which is the principle vector of *Candidatus* Liberibacter asiaticus, CLas, (Asia, Brazil and the United States). The Liberibacter bacteria can replicate in both the psyllid vector and host plants (Citrus and Solanaceous plants) [8]. Citrus tree infection with CLas causes tree decline, fruit loss and eventually tree death [9,10]. The resulting disease, Huanglongbing is the most severe-threat to global citrus production (a.k.a. Citrus Greening Disease) [11,12,13,14,15,16,17,18]. The Silverleaf whitefly, *Bemisia tabaci* (Gennadius 1889) (Figure 1B), is considered a species complex, with at least 40 morphologically indistinguishable species [19]. The whitefly is considered a supervector [20] transmitting hundreds of different viral pathogens to a wide variety of host plants that includes over 300 food crops, ornamentals and weeds [21,22,23,24,25]. The glassy-winged sharpshooter leafhopper, *Homalodisca vitripennis* (Germar, 1821) [26] (Figure 1C), is a vector of *Xylella fastidiosa* plant-infecting bacterium that is a xylem-limited plant pathogen that leads to decline, fruit loss and plant death of fruit trees, grapevines, olive trees and woody ornamental plants. *Xylella fastidiosa* infection and pathology in grapevines is called Pierce’s Disease of grapevines; infection in citrus is Citrus Variegated Chlorosis, symptoms of dried leaves, withered or no fruit, often called ‘Scorch-like’ disease across fruit trees, olive trees, nut crops and woody ornamentals [27,28,29,30,31,32,33].

The global distribution for each insect vector can be found at the Open Source: Invasive Species Compendium (CABI) (https://www.cabi.org/isc/, accessed 15 July 2021) with CABI-Distribution-Maps, for each vector, distribution maps and their economic importance as insect vectors. The Asian Citrus Psyllid (https://www.cabi.org/isc/datasheet/18615#todistribution, accessed 22 June 2021); Whitefly, *Bemisia tabaci* (https://www.cabi.org/isc/datasheet/8927#toDistributionMaps, accessed 15 July 2021); and Glassy-winged sharpshooter, *Homalodisca vitripennis,* (https://www.cabi.org/isc/datasheet/27561#toDistributionMaps, accessed 22 June 2021). More information on the Taxonomy, Biology, Vector status and control treatments can be found in the vector datasheets for these three insect vectors: *Diaphorina citri*, Asian citrus psyllid (https://www.cabi.org/isc/datasheet/18615, accessed 22 June 2021); *Bemisia tabaci*, (https://www.cabi.org/isc/datasheet/8925), and *H. vitripennis* (https://www.cabi.org/isc/datasheet/27561) (accessed 22 June 2021). (Figure S1. Global Distribution Maps; and S7 Report: Gene Data-mining from De Novo Genomes (Psyllid, Whitefly, Leafhopper), Supplemental Materials).

Advances in genomics, bioinformatics and other –Omics have created a paradigm shift from tritrophic interactions, to one that uses a multi-level, multi-organism ‘Systems Biology’ approach to crop-pathosystems [34]. Systems Biology depends upon the production and examination of the largest amount of data possible. This includes the entire fauna of organisms in the system, the environment, chemistry and cultural practices that can affect the desired outcome (i.e., sustainable crop production), AgriVectors.org [35]. Implementing a larger view of pathogen/vector/plant interactions can also result in rapid development of solutions to manage the insect vector(s), and crop pandemics, thus reducing human mortality due to food shortages and starvation [7,36,37]. The biology of hemipteran vector species, such as aphids, psyllids, leafhoppers, planthoppers and whiteflies, include members with broad host ranges, with high reproductive rates, that can rapidly develop insecticide resistance, and often they have the capacity to transmit multiple types and species of pathogens [4,5,6,29,31,37,38,39,40,41]. Attempts to suppress insect populations with chemical insecticides is standard practice but often only provides a short-term solution. Continuous spray treatments eventually lead to a long-term problem of insecticide resistance development [42,43,44,45,46,47,48]. Emerging gene-targeting technologies, such as RNA interference, RNAi, provide new strategies to develop more effective and insect specific biopesticides to address some of these problems [49,50,51,52,53,54,55,56,57,58,59,60]. An RNAi biopesticide provides specific gene-targeting of the RNA’s inside of insect or pathogen reducing the potential negative impacts on non-target species, while providing safer pest control [61,62,63,64,65,66,67]. For a brief history of RNAi see Sen and Blau (2006) [68]. Reviews on the breakthroughs in applications and delivery methods of dsRNA for RNAi in insects include, but are not limited to the following (e.g., and references contained therein: [49,51,52,53,54,57,69,70,71,72,73,74,75,76,77]. The RNAi mechanism is a natural cell process that occurs in almost all eukaryotes [78,79]. The RNAi mechanism is used to regulate cellular translational suppression and as a defense mechanism against viral infection and mobilized transposable elements by Ding (2010) [80]. The process is triggered by the presence of double stranded RNA, dsRNA, which is bound by an RNase III enzyme called *Dicer.* Upon binding the *Dicer* enzyme cleaves the dsRNA into short fragments that are 21–25 nucleotides long. These are called small interfering RNA’s (siRNA) [81]. The siRNAs are incorporated into the RNA induced silencing complex (RISC), which unwinds the siRNA, and uses the now single-RNA ‘guide strand’ within the Argonaute protein of the RISC to mediate complementary recognition, by Watson-and-Crick base-pairing, resulting in the cleavage of the mRNA, thus preventing protein translation [82].

Other types of RNA targeting aptamers, which have also used modified nucleotides include antisense oligonucleotides such as FANA’s [83,84,85,86], morpholino’s [34,87,88,89,90,91,92,93] and other aptamers (i.e., microRNA, single-stranded RNA, etc.) used for medical and agricultural applications [49,85,94,95,96,97,98,99,100,101,102,103,104]. While RNAi continues to be gained attention there are still some ‘gaps’ that need to be optimized to produce a biopesticide that can be competitive with commercialized chemical products. The two most common issues are improving the stability after field application, and to improve RNAi activity after ingestion by insects [1,55,56,73,95,105].

Studies of RNAi knockdown in the Asian citrus psyllid, across all life stages are reviewed by Yu and Killiny (2020) [106]. The earliest RNAi in the Asian citrus psyllid and the glassy-winged sharpshooter sequenced the *Arginine kinase*, to produce *AK-* dsRNA’s that were evaluated between species showing RNAi specificity [107], following studies validated the effects of *AK, actin, SOD, Trehalase* and *cactin* -dsRNA’s [34,50,87,108,109,110,111]. A partial list of genes targeted in other RNAi studies in psyllid have reported knockdown of: *trehalase* and *trehalose-6-phosphate synthase* gene [112]; *Superoxide dismutase* [113]; the abnormal normal *wing disc* gene [114]; injection and feeding of CYP-dsRNA’s to suppress insecticide resistance [115,116]; suppression of two glutathione S-transferase genes, DcGSTe2 and DcGSTd1, increased psyllid susceptibility to pesticides [117]; and carboxylesterases to increase nymph mortality [118]. The strong RNAi response to dsRNA triggers shows that RNAi has the potential to be used to manage psyllid pests.

RNAi-based studies in whiteflies show significant knockdown of a wide set of gene targets, with reviews by Kaur et al. (2020) [119], Luo et al. (2017) [24] and others (e.g., and references therein: [120,121,122,123,124,125,126,127,128]. The earliest RNAi in whitefly, *B. tabaci*, used dsRNA’s of *actin*, *BtSnap* and *chickadee* [129]. Many other studies have been conducted with only a few examples give here. RNAi knockdown of juvenile hormone esterase in *B. tabaci* that caused effects on adults and progeny [121]; suppression of whitefly osmoregulators [130]; the suppression of whitefly fed on tobacco expressing dsRNA for whitefly v-ATPase A gene [131]; and knockdown of the gut genes using specific dsRNA’s [132]. Functional gene studies also used RNAi to characterize the *doublesex* gene in adult *B. tabaci* [133]. RNAi-based studies in whitefly show that whitefly have a roust RNAi response to dsRNA, and that RNAi biopesticides could be used to manage whitefly pests.

RNAi-based studies in leafhoppers, specifically for *H. vitripennis*, the glassy-winged sharpshooter leafhopper have not been as prolific. Some of the earliest studies include feeding *Arginine kinase*-dsRNA through treated plants and cut plant tissues (citrus, grapevine, okra) [107,108,110] and using injections of *actin*-dsRNA [134,135]. Greater attention by researchers has been on the planthoppers that are pests of grain crops (rice, maize, wheat) [136]. The majority of leafhoppers and planthoppers of economic importance are in the same taxonomic suborder Auchenorrhyncha [137]. Reviews on RNAi in hemipterans show increasing research and development across a growing number of these important leafhopper and planthopper vectors [123,138,139]. Examples include the Green Rice Leafhopper, *Nephotettix cincticeps;* laccase-2 gene knockdown in first instar nymphs [140]. The cotton leafhopper, *Amrasca biguttula biguttula* (Ishida) functional genomic studies that evaluated stress effects on 15 common housekeeping genes (*Tub, B-Tubulin, Elongation-alpha*, *GADPH*, *UbiCV*, *RP13, Ubiq*, *G3PD, VATPase*, *Actin 18s 28s*, *TATA*, *ETF*, *SOD* and *Cytolytic actin*) [141]. RNAi-based studies in the brown planthopper, *Nilaparvata lugens* Stål, show knockdown of vacuolar ATP synthase subunit-E (*V-ATPase-E*) dsRNA in sucrose solutions, and trehalose phosphate synthase, fed to *N. lugens* [142,143]. The knockdown of midgut genes, the *hexose transporter* gene, the *carboxypeptidase* gene, and the *trypsin-like serine protease* gene in *N. lugens* [144]. A microRNA and dsRNA targeting *chitin synthase A* in *N. lugens* [145,146].

Even though there are many published studies using exogenous RNAi to significantly reduce insect vectors, including many reports of gene target suppression after ingestion of dsRNA by most plant-feeding hemipterans, such as the examples above, and in the following reviews (e.g., and references therein: [49,57,58,123,138,139], there is a need to optimize the RNAi activity to maximize efficacy, so that the concentration and cost per treatment can be reduced.

Therefore, research was conducted to optimize the RNAi activity by improving the dsRNA triggers. Presented is a comparative evaluation of unmodified dsRNA *versus* dsRNA modified with pyrimidine nucleotides, 2′-F-Uridine and 2′-F-Cytosine, to demonstrate improved RNAi activity of exogenously applied dsRNA for the suppression of mRNA targets (*Soluble Trehalase*, *Syntaxin 1A* and *Cactin*), evaluated using three hemipteran vectors (Asian citrus psyllid, Silverleaf Whitefly and Glassy-Winged Sharpshooter Leafhopper).

## 2. Rationale Gene Target Selection

Insect genomes and transcriptomes provide the foundational information needed to design effective RNAi pest management. The development and use of the psyllid, whitefly and leafhopper genomes were multi-institutional efforts that produced open-source data sets and are described in the Supplemental Material (S7. Gene Data-mining from De Novo Genomes (Psyllid, Whitefly, Leafhopper)). Gene targets in this study were selected after in-depth literature searches from studies reporting genes with a critical function in embryo and larvae development from studies in mammals [147,148,149], nematode, *Caenorhabditis elegans* (e.g., and references therein: [150,151] and insects, such as *Drosophila*; and *Tribolium* (e.g., and references therein: [71,149,152,153,154,155]. The sequences were assembled from de novo genomes, transcriptomes or from the Sequence Read Archive (SRA) deposited by the Genome Consortiums of each insect (NCBI database) https://www.ncbi.nlm.nih.gov/sra/docs/ (accessed on 8 July 2021)) followed with validation by resequencing from each insect vector. (S7 in Supplemental Materials).

### 2.1. Soluble Trehalase

Soluble *Trehalase*—Research has shown that *Trehalase* has critical functions in hemipteran development and survival [142,155,156,157]. When *Trehalase* is suppressed then the synthesis of *Trehalose* is reduced and there is an increase in psyllid mortality [112], with similar reports in planthoppers [158] and other invertebrates [159,160]. *Trehalose* is the main blood sugar of insects, and the enzyme trehalase is involved in energy metabolism and regulates trehalose levels in cells. The two forms of *Trehalase* in psyllid (soluble and membrane bound) and their corresponding genes were identified from data mining the *D. citri* pathway network dataset, of the *Diaci_2.0 genome* and the official gene set (OGS-v2, 2019) (open access at: www.citrusgreening.org) [161,162,163,164,165,166]. The soluble *Trehalase-1b,* mRNA sequence, *Trehalase* (EC:3.2.1.28) and the regions selected for dsRNA production are shown in the Supplemental Materials (Figure S2). Phylogenetic trees for the putative proteins for the psyllid *Trehalase*, and *Syntaxin 1A* in psyllid, *D. citri* (MCOT10768.0.MT) and whitefly, *B. tabaci* (KC161217.1), and the *Cactin* sequence (*D. citri* (XM_008474513.2)*, B. tabaci* (XM_019060862.1) *and H. vitripennis (HVIT015866-RA)* were compared using BLASTn, BLASTx and BLASTp to the Order: Hemiptera, online at NCBI (June 10, 2021). Phylogenetic trees for *Trehalase*-1 in *Diaphorina citri*, (Figure S3) and *Syntaxin 1A* (*D. citri* and *B. tabaci)* (Figure S4), and *Cactin* (*D. citri,* Figure S5) were generated to other hemipteran insects, (NCBI, BLASTx TreeView) [167,168,169,170] in Supplemental Materials.

### 2.2. Cactin

*Cactin*—A poly(A) RNA-binding conserved protein that was first described to have important functions in *Drosophila melanogaster*, by Lin et al. (2000) [171]. *Cactin* interacts with the ‘kappa-light-chain-enhancer’ IkappaB protein *Cactus* and modulates its function [171]. The IκB inhibitor *Cactus* functions in promoting dorsal nuclear localization and activity in the insect embryo for proper development [172]. RNAi loss-of-function studies have shown that *cactin* is essential for *Wingless* and *Int-1* (*Wnt*) signaling important for normal embryonic and larval development, thus *cactin* is considered a good RNAi target for pest control [173,174]. In the beetles *Tribolium castaneum*, and *Diabrotica virgifera virgifera,* Bingsohn et al. (2017) [175] reported 100% mortality in all life stages when *cactin* was silenced. Over 90 other significant RNAi targets have been proposed from the iBeetle large-scale RNAi screening effort for insect development and physiology [153,154]. Similarly, over 100 RNAi targets have been validated in the nematode *C. elegans* (see, [176]; WormBase: Nematode Information Resource, https://www.wormbase.org, accessed 6 June 2021).

### 2.3. Syntaxin 1A

*Syntaxin 1A—*Interacts with multiple exocytic proteins to regulate neurotransmitter release, [177,178,179], and modulates sexual maturity rates and progeny egg size related to phase changes in locusts [180], while providing guidance in a conserved role for pre- and post-commissural midline axonal formation in flies and other insects [152,181,182]. In the hemipterans we identify multiple variants of *Syntaxin 1A* (1–4) supporting the findings of other studies in dipterans, *Drosophila* [179] that reported the *Syntaxin 1A* subfamily to consists of only two genes that function as the t-SNARE in synaptic vesicle fusion (dStx1 and dStx4). This is different from the human *Syntaxin 1A* subfamily having 12 genes, with five that function as the t-SNARE (Stx1, Stx2, Stx3, Stx4 and Stx11) (see, e.g., [149] and references therein).

## 3. Optimizing *dsRNA*

Optimizing *dsRNA*—Examination of the knowledge gained from over 30 years of research on chemical modified nucleotides and their improved characteristics for drug development, led to the conclusion that these modifications could help to optimize RNAi activity in insects (e.g., reviews on current advances and references therein: [85,94,95,96,97,98,101,183,184,185,186,187,188]). The benefits of incorporating 2′-F- designs in dsRNA for RNAi ‘triggers’ are well documented to improve the stability and activity [49,83,85,101,102,104,186]. Research experiments with nucleobase, ribose or phosphate modifications reviewed by Chernikov et al. (2019) [95] clearly shows the potential effects that each type of modification has on the properties, their sensitivity to ribonucleases and their interactions with the RNAi recognition and processing enzymes. A list of the modifications that increase resistance to enzymatic degradation and that increase binding affinity to produce improved RNAi activity are also found in the review by Glazier et al. (2020) [189]. An additional benefit of RNAi biopesticides is their specific nature: their capacity to reduce an insect pest, while not negatively affecting non-target species, such as pollinators, predators and parasitoids [49,53,54,56,57,61,66,67,76,190,191,192]. Even though RNAi strategies, that use dsRNA triggers are effective at targeting viruses, insect pests and vectors of plant-infecting bacteria, RNAi-based triggers are not effective at suppression of bacteria [49]. To reduce some of the negative impacts from chemical pesticides and herbicides, there has been an increasing shift to develop biopesticides, such as RNAi treatments that will degrade rapidly in the environment [61,193,194,195,196]. As the world population continues to increase food security threats from insect vectors will need to develop more effective biopesticides for the future of sustainable agriculture [54,57,61,75,76,104,105,197,198].

In the present study, we attempted to optimize dsRNA triggers used to reduce three hemipteran insect vectors. The study compared dsRNA triggers made with unmodified nucleotides, with dsRNA’s that incorporates modified 2′F- pyrimidines. The use of the modified pyrimidines consistently improved transcript suppression, with significant increase of insect mortalities after ingestion from treated plants and plant tissues.

## 4. Results

### 4.1. RNAi Suppression of Psyllids Feeding on Citrus Seedling Trees

#### 4.1.1. Pre-Trial Bioassay to Determine dsRNA Activity

The dsRNA triggers were made to the soluble *Trehalase mRNA in D. citri* and applied as exogenous topical sprays to citrus seedlings at 70 µg dsRNA per plant and provided for a 15-d feeding access period.

#### 4.1.2. Evaluation of dsRNA’s to Induce RNAi Activity in Asian Citrus Psyllid

**Figure 2:**
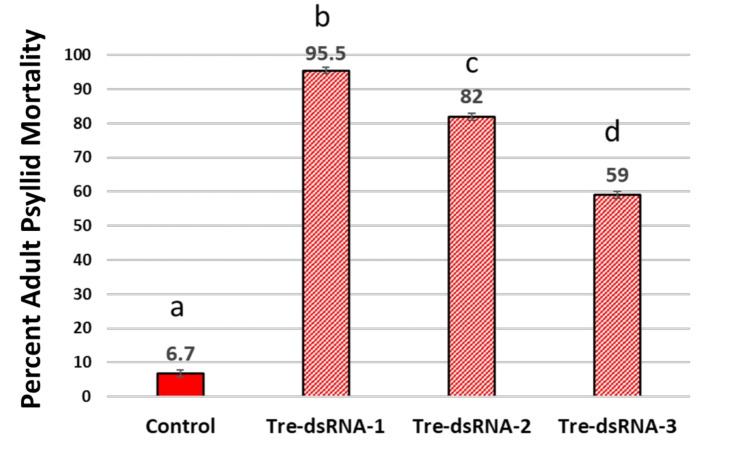
Adult psyllid mortality at 15-d-feeding access on *Trehalase*-dsRNA treated citrus cuttings. RNAi activity comparison of three dsRNA triggers made to different regions of the soluble *Trehalase* mRNA in the Asian citrus psyllid. There was statistically significant differences in adult psyllid mortality between group means of treatments as determined with one-way ANOVA (*p* < 0.05). Statistical significance of means *post-hoc* Tukey (*p* ≤ 0.01). Error bars represent ±SE of mean (n = 8) calculated on eight biological replicates, with three technical replicates. Experiment repeated twice. Treatments means with the same letters were not significantly different.

Pre-trial screening and evaluations of three unmodified, *Trehalase* dsRNA triggers to different regions of the soluble, *Trehalase-1* mRNA in *D. citri,* identified dsRNA triggers (Tre-1, Tre-2, Tre-3) that induced a strong RNAi response, resulting in significantly increased mortality from 59%, to 82%, to 95.5%, respectively (Figure 2). Feeding bioassays with new growth citrus cuttings are a natural system for psyllid feeding from citrus phloem, but also enable rapid delivery of the dsRNA into the plant tissue for evaluations. Method was described as an *inPlanta* System, iPS [108].

#### 4.1.3. Unmodified dsRNA Triggers and Adult Psyllid Mortality

RNAi activity comparison of three dsRNA to different regions of the *trehalase*-mRNA fed to the Asian citrus psyllid (Figure 2). There was statistically significant differences between group means per sample, for adult psyllid mortality as determined by one-way ANOVA, F(3, 140) = 1767.05, *p* < 0.01, α = 0.05, η^2^ = 0.97. Thus, the null hypothesis of “no significant differences” between group means was rejected, and 97% of the variance in mortality was accounted for by treatment group. Pairwise comparison of means *post-hoc* with Tukey (HSD) (*p* < 0.05). Psyllid mortality evaluated after a 15-d feeding access period showed significant differences with the greatest mortality observed in the *Trehalase-*dsRNA-1 treatment. This dsRNA trigger was 468 nt and targeted the middle region of the mRNA, which produced an average mortality of 95.5% (±SE 0.55). Plants treated with *Trehalase*-dsRNA-2 produced 82% mortality (±SE 0.32) and targeted the most 5′ region of the mRNA, approx. 106 nt from the ATG start codon. The *Trehalase*-dsRNA-3 averaged 59% mortality (±SE 0.43) targeting the most 3′ region of the mRNA. Mortality in the control treatment averaged 6.2% (±SE 0.10) (Figure 2). There was statistically significant differences in adult psyllid mortality between group means of treatments as determined by one-way ANOVA, F(3: 20) = 252.44, *p* < 0.01; α = 0.05, η^2^ = 0.97. Thus, the null hypothesis of ‘no differences’ between means was rejected, and 97% of the variance in mortality was accounted for by treatment. Statistical significance of means post-hoc Tukey (*p* ≤ 0.01). Error bars represent ± SE of mean (n = 8) calculated on eight biological replicates, with three technical replicates. Experiment repeated twice. Pre-trial evaluations identified the most active dsRNA for use in subsequent feeding bioassays (Sequences in Figure S2, Supplemental Material).

## 5. Comparison of dsRNA to Soaps as Insecticidal Agents against Psyllid

### 5.1. Background

Common use of insecticidal soaps (M-PEDE or Safer Insecticidal Soap Concentrate) for insect control are approved for commercial use, homeowners and organic growers [199]. Product sources for M–Pede (Gowan Company, LLC, Yuma, AZ, USA) and Safer^®^Insecticidal Soap Concentrate (Woodstream Corporation, Lititz, PA, USA). Sprays of M-Pede™ or Safer Soap™, at concentrations of 2% *v*/*v* in water on citrus trees infested with psyllid adults and nymphs was only effective with repeated (>3) applications to produce 100% mortality. The repeated applications are to contact nymphs that emerge from eggs that were protected during the initial treatment. Direct sprays of soap solutions (0.8–2% in water) of either M-Pede or Safer Insecticidal Soap were acutely toxic to psyllid adults and nymphs (regardless of gender). However, the insecticidal soaps were not toxic to eggs at rates of up to 2% [199]. Residues of the soaps were not effective at reducing adult psyllids, even when the concentrations were increased to 4% [199]. Since the soaps caused some mortality effects of early nymphs upon hatching, we chose to compare Safer Insecticidal Soap Concentrate, at 5% in water with a dsRNA-spray treatment.

### 5.2. Psyllid Survival over a 24 Day-Feeding Access Period on Trehalase-dsRNA Treated Citrus Seedlings

Treatment solutions were control water; *Trehalase*-dsRNA, and a 5% Insecticidal Soap exogenously applied as aqueous sprays onto potted citrus seedlings.

Oviposition Number of Eggs per Plant, per Flush, per Treatment. The mean number of eggs oviposited under these conditions per seedling across each treatment were not significantly different, (One-way ANOVA, *p =* 0.664761, α = 0.05). Average eggs per plant in each treatment were: 42.1(±SE 0.86) eggs in Control plants, 39.4 (±SE 2.32) in the 5% Soap treatment and 41.3 (±SE 2.40) in the *Trehalase*-dsRNA treatment (n = 9) (Table S2) in Supplementary Material.

**Figure 3:**
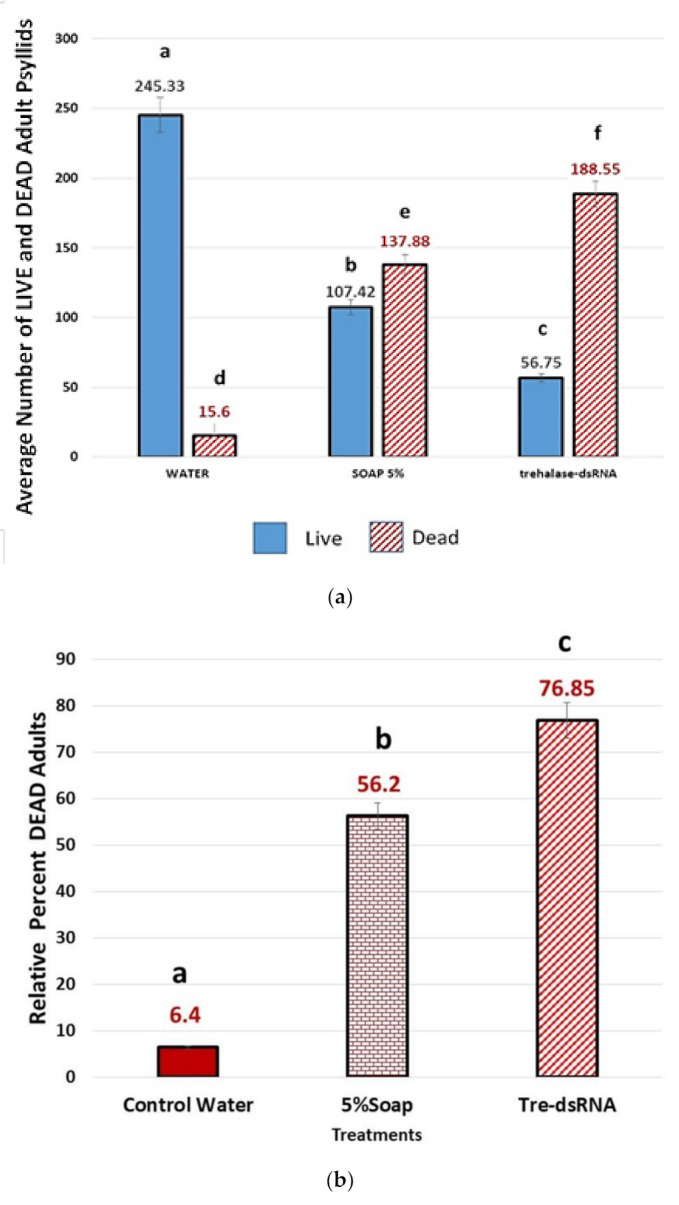
Average number of surviving adult psyllids, at the end of a 24-d-feeding access period on potted citrus seedlings (**a**). Three treatments: Control Water, 5% Insecticidal soap and *Trehalase*-dsRNA. There were statistically significant differences between group means within live and dead as determined by one-way ANOVA, followed with *post-hoc* analyses Tukey (*p* < 0.05). The relative percent of Dead shown in (**b**), Tukey (*p* < 0.01). Error bars represent the ± SE of the mean (n = 12) from 12 biological replicates with four technical replicates, from three independent experiments. Bars with the same letter within group, ‘Live’ or ‘Dead’ were not significantly different.

Numbers of Adults per Treatment. Analyses of the numbers of surviving psyllids, representing adults at the end of the 24-d trial within each treatment showed there were statistically significant differences between group means as determined by One-way ANOVA, F(2, 33) = 537.93, *p* < 0.01, α = 0.05, η^2^ = 0.97. Thus, the null hypothesis of ‘no significant differences’ between group means was rejected, and 97% of the variance in mortality was accounted for by treatment. Means separation with post-hoc Tukey (*p* ≤ 0.01). For Live adults in each treatment, the water control averaged 245.33 (±SEM 7.38) Live, the 5% Insecticidal Soap treatment averaged 107.42 (±SEM 2.36) Live, compared to significantly fewer live adult psyllids in the *Trehalase*-dsRNA-treatment, (56.75, ±SEM 0.81) (*p* < 0.01) (Figure 3A). For mortality, the relative counts of dead adult psyllids per treatment were 15.6 (±SEM 5.85) for water control, and 137.9 (±SEM 2.36) in the 5% Insecticidal Soap treatment, with a significantly greater number of Dead adult psyllids, 188.6 (±SEM 0.81) in the *Trehalase*-dsRNA treatment. There were statistical significant differences between group means for dead as determined by one-way ANOVA, F(2, 33) = 537.93, *p* < 0.01, α = 0.05, η^2^ = 0.97. Thus, the null hypothesis of ‘no significant differences’ between group means was rejected, and 97% of the variance in mortality was accounted for by treatment. Pairwise comparison of means post-hoc Tukey (*p* ≤ 0.01). The overall mean percent of Dead psyllids per treatment shown in (Figure 3B). The null hypothesis was there would be no difference in the numbers of adults produced between in each treatment. Since the dsRNA treated groups would have increasing mortality, the Total number of live psyllids on the control trees were used as the expected number to be alive at the end of the trials. Thus, the relative percent mortality was calculated within each category using the Total Number of Live psyllids in the Control groups (n = 2944), being used to normalize the maximum number (100% of insects possible) from each treatment under the experimental design. This accounts for randomness of molting success in each psyllid population on each plant. The Total Number of Dead in each treatment was the numerator, × 100.

**Figure 4:**
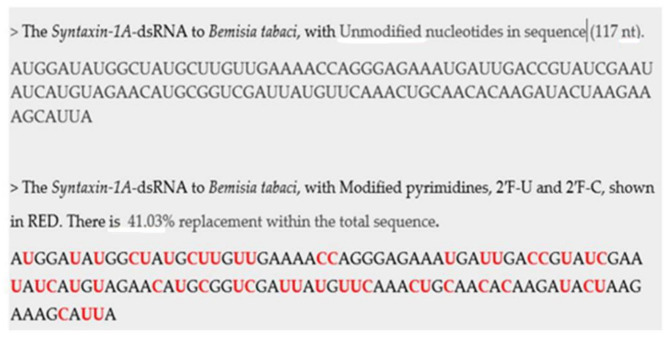
Example of unmodified dsRNA and modified pyrimidines, 2′F-C and 2′F-U dsRNA triggers to *Syntaxin 1A*, in the whitefly *Bemisia tabaci.* The percent of modified pyrimidines- (2’-F-C and 2’-F-U), was 41.03% of the total sequence of 117 nucleotides, but efficacy of replacement ranged from 30 to 55%. The modified, noncanonical, 2′-F-Cytosine and 2′-F-Uracil dsRNA was synthesized using the Lucigen^®^ DuraScribe^®^ T7 Transcription Kit (Cat. No. DS010925).

Figure 4, provides an example showing the nucleotide sequence for the *Syntaxin-1A-*dsRNA trigger. The same dsRNA sequence showing one with the modified pyrimidines in place, and the unmodified dsRNA.

### 5.3. RNAi with 2′-F- Modified Pyrimidines in Cactin-dsRNA Increased Psyllid Mortality

#### 5.3.1. RNAi Treatment Inhibited Psyllid Nymph Development to Adulthood

**Figure 5:**
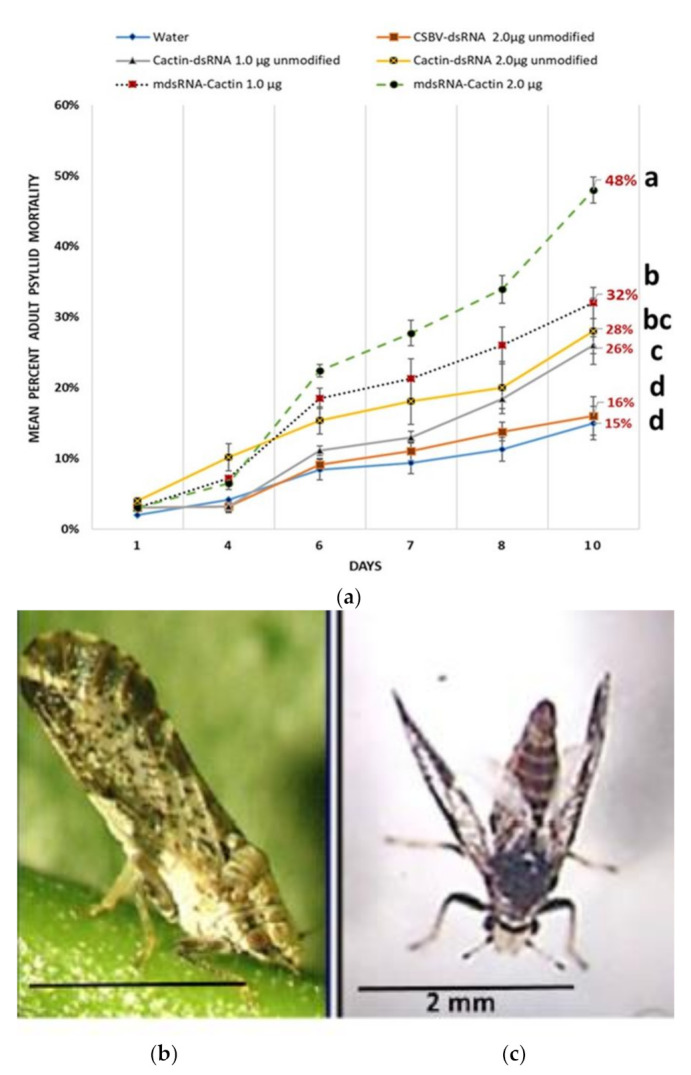
Mean Percent Psyllid Mortality. Analyses of psyllid Nymphs, 4th instar given a 10-d feeding access period on citrus cuttings treated with either modified or unmodified *Cactin*-dsRNA (**a**), compared to the CSBV-dsRNA control(2 µg concentration) and a blank water control, identified statistically significant differences between group means described by one-way ANOVA, followed by pairwise comparison of means with *post-hoc* Tukey (*p* < 0.05). Error bars represent the ±SE of the mean (n = 9) as determined from nine biological replicates, three technical replicates. Experiments repeated three times. Percentages with the same letter were not significantly different. (**b**) Psyllid adult feeding on citrus leaf midrib. Wings are held together over body. (**c**) Psyllid adult after an 8-d-feeding access period on citrus cuttings from Cactin-dsRNA treated citrus seedlings. Wings appear ‘Flared’ producing a phenotype that has trouble walking, feeding and died earlier than controls. Bar in figures = 2 mm.

The relative percent of psyllid mortality of 4th instars over a 10-d-feeding access period on citrus cuttings treated with *cactin*-dsRNA is shown in (Figure 5). There was a significant difference in psyllid mortality resulting in fewer adult psyllid on the dsRNA treatments at the end of the 10-d feeding period, as determined by one-way ANOVA, F(4, 40) = 515.41, *p-*value = 8.02 × 10^−34^, α = 0.05, η^2^ = 0.98. Thus, the null hypothesis of ‘no significant differences’ between group means was rejected, and 98% of the variance in mortality was accounted for by treatment. Pairwise comparison of means with post-hoc Tukey (*p* ≤ 0.05). On average the nymph’s eclosed as adults on day four. The psyllid adults showed significant increase in deformities and increased mortality. At the end of the 10-d feeding access period, the modified *cactin*-dsRNA, 2.0 µg treatment had significantly greater mortality (48%) compared to all other treatments (*p* < 0.01). There was no significant difference between the means of the 1.0 µg concentration of modified *cactin*-dsRNA treatment (32%) with the 2.0 µg concentration treatment of the unmodified dsRNA treatment (28%). The unmodified *cactin*-dsRNA mortalities were significantly different being less compared with their corresponding concentrations of the modified-dsRNA treatments, which had significantly greater mortality (26% and 32%, respectively) for the 1.0 µg dsRNA concentration treatments; and in the 2.0 µg concentrations, for unmodified (28%) compared to modified (48%) mortalities. The average mortality of the CSBV-dsRNA control (2 µg concentration) and water blank control were significantly lower than all other treatments (16% and 15%, respectively), Tukey (*p* < 0.05) (Figure 5A).

#### 5.3.2. Psyllid ‘Flared-Wing’ Adult Phenotypes Induced after Nymph Feeding on Cactin-dsRNA Treated Citrus Seedlings

Psyllid 4th instar nymphs were reared on *cactin*-dsRNA treated citrus cuttings, resulting in a significant reduction in adult psyllid emergence due to increased nymph mortality (26% to 48%). Adults that eclosed on the *cactin*-dsRNA treatments produced ‘Flared-winged’ phenotypes that could not fly, with a range of difficulty in walking and feeding (Figure 5C). The most severe deformed phenotypes were observed at the greater concentration of modified *cactin*-dsRNA, 2.0 µg. These psyllids presented ‘flared-forewings’, with slightly curled hindwings with reduced lifespan, dying on average within six days after eclosion to adult. Less severe phenotypes could still walk and feed, but could not jump or fly, and occurred to a much lesser extent in the unmodified *cactin*-dsRNA treatment (1 and 2 µg) (Figure 5B,C).

#### 5.3.3. Mean Adult Psyllid Mortality

**Figure 6:**
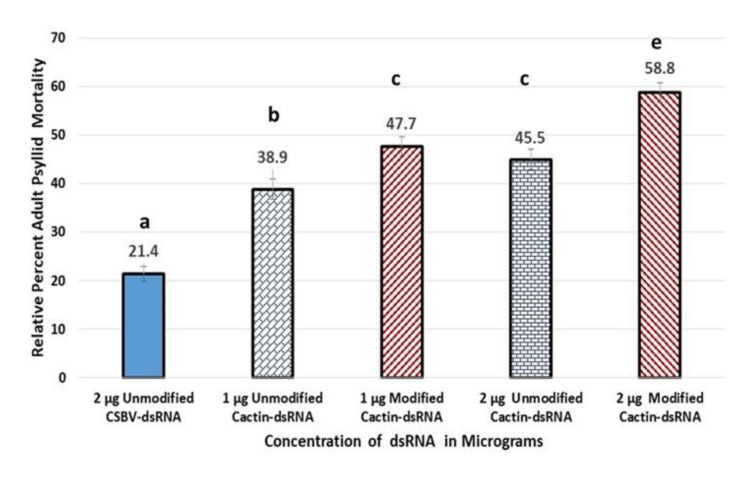
Percent Mean Psyllid Mortality at 8-d feeding access period on citrus cuttings treated with modified-pyrimidines in the dsRNA, or unmodified *cactin*-dsRNA at two concentrations. There was statistically significant differences between group means, as determine by one-way ANOVA, (*p* < 0.01), followed with means separation post-hoc Tukey (*p* ≤ 0.05). Error bars represent ±SE of the mean as determined from nine biological replicates (n = 9), with three technical replicates. Experiment repeated three times. Treatments with the same letter are not significantly different.

Mean Adult Psyllid Mortality after 8-d feeding access period on citrus cuttings treated with modified-pyrimidines *cactin-*dsRNA, or unmodified *Cactin*-dsRNA at two concentrations per plant (1 µg or 2 µg). There was statistical significant differences between group means, as determine by one-way ANOVA, F(4, 40) = 459, 73, *p-*value = 7.523 × 10^−33^, α = 0.05, η^2^ = 0.98. Thus, the null hypothesis of ‘no significant differences’ between group means was rejected, and 98% of the variance in mortality was accounted for by treatment. Means separation with post-hoc Tukey (*p* ≤ 0.05) (Figure 6). All the *cactin*-dsRNA treatments were significantly different from the CSBV-dsRNA control. The greatest mortality was observed in the modified *cactin*-dsRNA at the 2 µg concentration treatment (58.8%, ±SE 0.64), followed by the modified *cactin*-dsRNA at the 1µg concentration treatment (47.7%, ±SE 0.51) and the unmodified dsRNA 2 µg concentration treatment (45.5%, ±SE 075). The unmodified *cactin*-dsRNA 1 µg concentration treatment had the lowest mortality effects of the dsRNA treatments (38.9% ± SE 0.42), while the control dsRNA mortality was (21.4%, ±SE 0.66) (Figure 6).

#### 5.3.4. Fold-Change in Expression of Cactin mRNA in Adult Psyllids Given 6-d Feeding Access Period on Treated Citrus Seedlings

**Figure 7:**
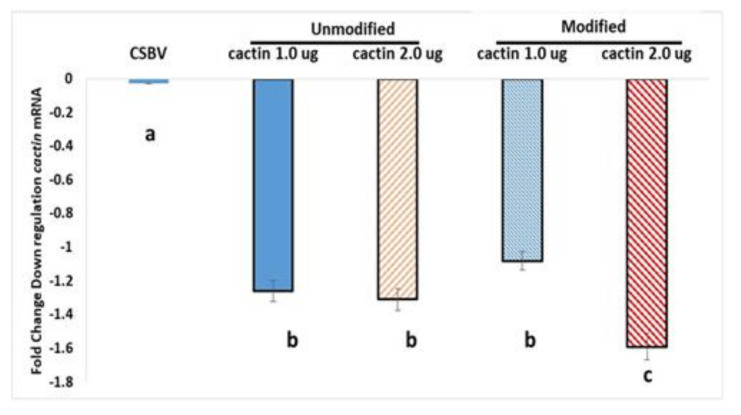
Fold-change in expression of *cactin* mRNA in adult psyllids compared to controls. There were statistically significant differences as determined by One-way ANOVA, followed by post-hoc Tukey (*p* < 0.05). All adult psyllids fed on *Cactin*-dsRNA treatments showed significant differences from the Controls, CSBV-dsRNA and water control (shown set to zero). The modified *cactin*-dsRNA at the 2 µg concentration treatment was significantly different from all other treatments. Bars with the same letters were not significantly different.

There were statistically significant differences between group means, as determine by One-way ANOVA on relative expression values of *cactin* mRNA, followed with post-hoc Tukey (*p* < 0.05). All psyllids fed on the *Cactin-*dsRNA treatments, unmodified or modified pyrimidines, were significantly different from the controls, CSBV-dsRNA and water control. The rt-qPCR data showed an average 1.26-fold downregulation in psyllids treated with unmodified, 1 µg *Cactin*-dsRNA and a 1.31-fold downregulation in psyllids treated with 2 µg *Cactin-*dsRNA unmodified, compared to water control (set to zero). The modified, dsRNA treatments produced a 1.1 fold downregulation in psyllids treated with 1 µg modified *cactin-*dsRNA and a 1.6 fold downregulation in psyllids treated with 2 µg modified-*cactin-*dsRNA. Statistical significance was observed between both modified *cactin*-dsRNA concentrations of 1 and 2 µg treatments, and the treatments were also significantly different for mortality (Figure 7). There were no significant differences between the unmodified treatment concentrations, 1 and 2 µg in expression, but the treatment mortalities (38.9% and 45.5%, respectively) were significantly different (Figure 6). Error bars represent the ±SE of the mean (n = 6), determined from six biological replicates. The experiment was repeated three time. Relative expression levels were calculated using the ∆∆Ct method [200] (Figure 7).

## 6. Mortality Increased in Leafhoppers Fed on Okra Seedlings Treated with Unmodified and Modified *cactin*-dsRNA

**Figure 8:**
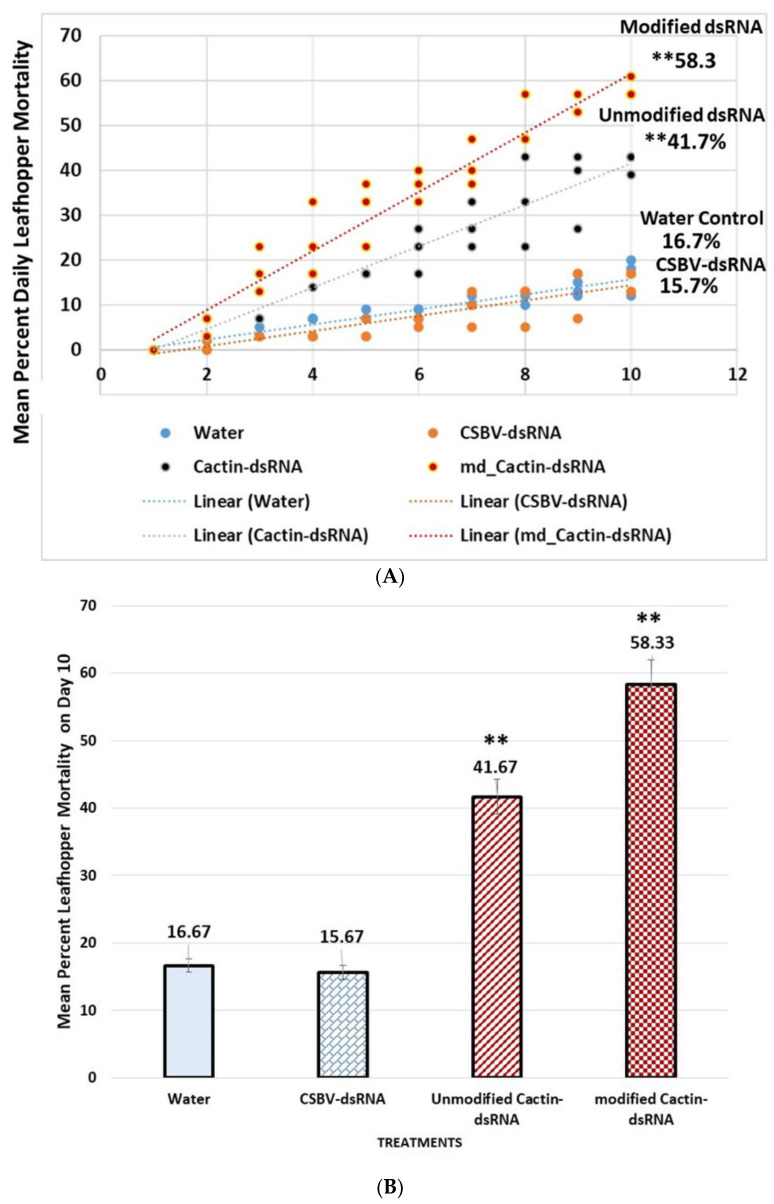
Percent Leafhopper Nymph Mortality. Analyses showed there was statistically significant differences between means of daily mortality over a 10-d feeding access period on treated okra cuttings (**A**), as determined by one-way ANOVA, with *post-hoc* Tukey (HSD) (*p* < 0.05). There were significant differences at day 10, between the modified *cactin*-dsRNA (58.33%, ±SE 3.61) and the unmodified dsRNA treatment (41.67% (±SE 2.58) and both controls (** *p* < 0.01). There was no significance between the water control (16.67%, ±SE 0.95) and the CSBV-dsRNA control (15.67%, ±SE 1.03) (*p* = 0.90) (**B**). Error bars represent ±SE of the means (n = 9) as determined from nine independent replicates, three technical replicates. Experiment repeated three times.

**Figure 9:**
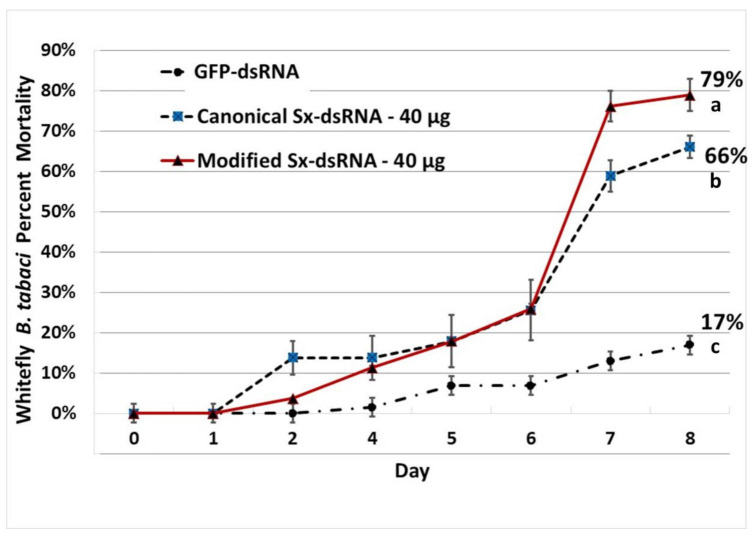
Average Percent Whitefly Mortality. Adult *B. tabaci* after an 8-d feeding access period on *Syntaxin*-dsRNA treated okra seedlings. Mortality was significantly greater in the modified *Syntaxin-*dsRNA treatment (79%) compared to the unmodified *Syntaxin*-dsRNA 66%) (*p* < 0.05), and to the GFP-dsRNA control (17%) (*p* < 0.01). One way ANOVA determined statistically significant differences between group means, followed by *post-hoc* Tukey (*p* < 0.01). Error bars represent ±SE of the means (n = 9) of three biological replicates, three technical replicates, from three independent experiments. Lines with different letters are significantly different.

Analyses of leafhopper mortality after feeding on dsRNA-treated okra seedlings showed there was statistically significant differences between means of daily mortality over a 10-d feeding access period (Figure 8A), as determined by one-way ANOVA, F(3, 116) = 25.98, *p* < 0.001, α = 0.05, η^2^ = 0.40. Pairwise comparison of means with Tukey (HSD) (*p* < 0.05). Mortality analyzed on the 10th day of feeding showed a statistically significant difference between group means (Figure 8B), as determined with Single Factor ANOVA, F(3, 8) = 154.1, *p* < 0.01, α = 0.05, η^2^ = 0.98, thus, the null hypothesis of ‘no difference’ between means was rejected, and 98% of the variance in mortality was attributed to treatment group. The post-hoc comparison of means with Tukey (HSD) (*p* < 0.05). Significant differences in mortality occurred between the modified *cactin*-dsRNA (58.3%, ±SE 3.61) and the unmodified dsRNA (41.7%, ±SE 2.58) treatments compared to the controls, CSBV-dsRNA (15.7%, ±SE 1.03) and the water control (16.7%, ±SE 0.95). Error bars represent ±SE of the means (n = 9) as determined from nine independent replicates, three technical replicates. Experiments were repeated three times. Relative expression levels were calculated using the ∆∆Ct method [200] (Figure 9).

## 7. Unmodified *Syntaxin*-dsRNA and Modified *Syntaxin*-dsRNA Reduce *Syntaxin 1A* mRNA Expression in Whitefly

### 7.1. The Relative Expression of Syntaxin

**Figure 10:**
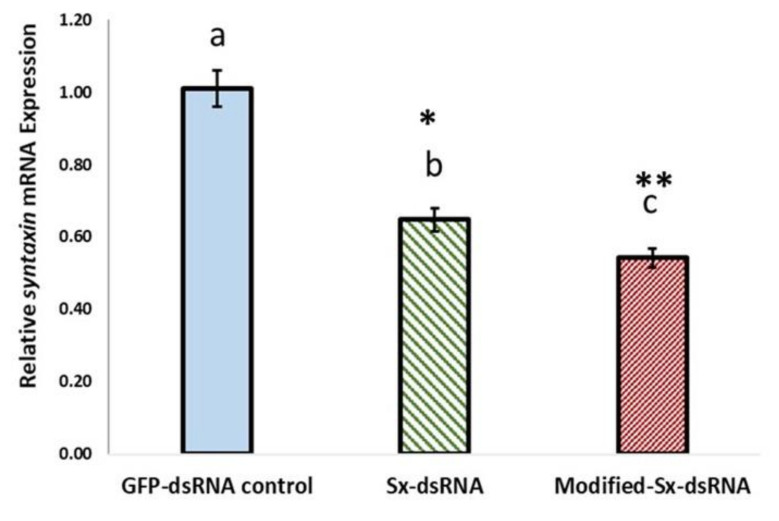
Mean fold-change in the expression of *Syntaxin-1A* mRNA in adult *Bemisia tabaci.* Whitefly were fed on okra seedlings treated with either modified- and unmodified- *Syntaxin*-dsRNA compared to unmodified GFP-dsRNA treated control. Whitefly *Syntaxin-1A* expression was significantly reduced when whitefly fed on plants treated with either *Syntaxin*-dsRNA, showing a change of 1.6 (±SE 0.1) and 1.9 (±SE 0.08) fold down-regulation, respectively. The modified *Syntaxin*-dsRNA treatment was significantly different from the unmodified *Syntaxin-*dsRNA (** *p* < 0.01), and both were significantly different from the GFP-dsRNA control (* *p* < 0.05). The GFP-dsRNA *Syntaxin* mRNA expression level set to 1.00. Error Bars represent the ±SE of means, (n = 9), from three biological replicates and three independent experiments. Bars with different letters are significantly different.

The relative expression of *Syntaxin*-*1A* mRNA in whitefly fed on *Syntaxin*-dsRNA treated okra seedlings with unmodified nucleotides, or modified pyrimidines dsRNA triggers was compared with the control GFP-dsRNA treatment. There were statistically significant differences between group means, as determined by one-way ANOVA, F(2, 24) = 29.18, *p* = 3.75E-07, α = 0.05, ƞ^2^ = 0.71. Pairwise comparisons post-hoc analyses of means with Tukey (HSD) (* *p* < 0.05, ** *p* < 0.01). Biological replicates (n = 27), three technical replicates, from three experimental trials. Bars with the same letter were not significantly different. Error bars represent the standard error of mean for nine biological replicates (n = 9) each with three technical replicates from three independent experiments. Relative expression calculated using the 2−∆∆Ct method [200] (Figure 10).

### 7.2. Evaluating Unmodified Nucleotides- vs. Modified Pyrimidines in dsRNA for Improved RNAi Activity in Bemisia Tabaci

Whitefly nymphs given a 9-d feeding access period on tomato cuttings treated with either unmodified or modified dsRNA triggers targeting the *Syntaxin* 1A transcript in the whiteflies, showed statistically significant differences between group means for survival as determined by One-way ANOVA, with pairwise comparison post-hoc with Tukey (*p* < 0.05).

**Figure 11:**
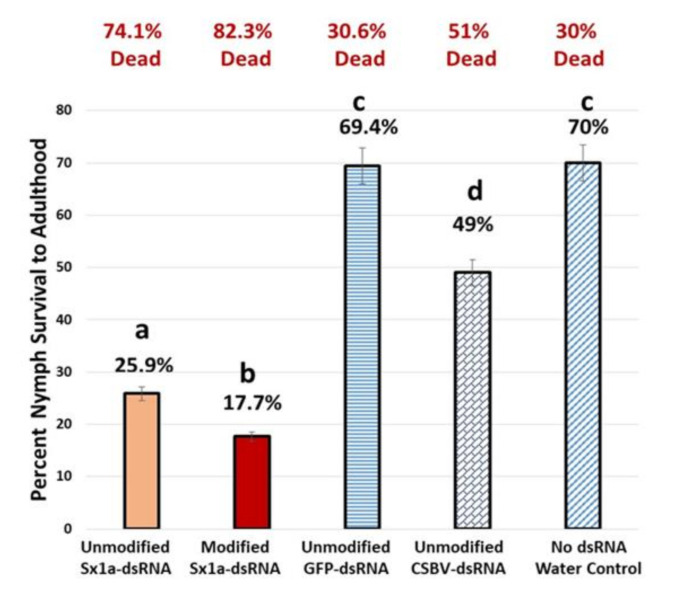
Evaluating unmodified nucleotides- vs. modified pyrimidines in dsRNA for improved RNAi activity in *Bemisia tabaci*. Significantly fewer whitefly nymphs completed development to adulthood on the tomato plants treated with modified (17.7%) and unmodified (25.9%) *Syntaxin*-dsRNA, compared to all controls (GFP-dsRNA 69.4%), CSBV-dsRNA (49%) and water control, No dsRNA (70%). Bars with ±SE of the mean, (n = 25), with six biological replicates, with three technical replicates. Experiment repeated three times. Bars with different letters are significantly different (*p* < 0.05).

Results showed that in the modified *Syntaxin*-dsRNA treatment there was a survival of 17.7% of whitefly nymphs that developed into adults at 9 days post-treatment (end of experiment due to plant size). In the unmodified *Syntaxin*-dsRNA treatment there was a 25.9% survival of whitefly nymphs that developed into adults (Figure 11). This translated into 82.3% whitefly mortality among developing nymphs feeding on the modified *Syntaxin*-dsRNA treatment, and 74.1% mortality of nymphs feeding on the unmodified *Syntaxin* dsRNA treated plants. The experimental condition mortality is demonstrated in three control treatments, which were found to have significantly greater percentages of survival (49–70%), (representing significantly lower mortality) with average mortality ranges from 30–51%.

## 8. Discussion

The results from this study are a proof-of-concept for optimization of dsRNA triggers by incorporation of modified pyrimidines 2′F-U, and 2′F-C, at a substitution of 30 to 55% to improve the RNAi activity, measured as a significant increase in insect mortality. As an indication of the dsRNA cellular activity a fold change >0.5 in gene expression from the internal control was used to confirm dsRNA activity [201] and effectiveness, when feeding bioassays from plants and plant tissues were employed. The improved mortality from the modified dsRNA may be due in part to a stronger binding efficiency. Glazier et al. (2020) [189] reported that the 2′-F transcripts were more sensitive to the change in ion charge from cellular salt concentration than the binding by unmodified 2′-OH RNA transcripts in mammal cell culture. They propose that interaction with viral transcripts may have a more ionic character for the modified 2′-F RNAs than for unmodified 2′-OH RNAs. Beyond the improved binding efficiency, the increase in mortality may also be in-part from release of fluorine ions that increase cell toxicity in the insect as the concentrations increase over time [201,202,203,204]. A report from the U.S. Food and Drug Administration, FDA, showed that 45% of small molecule drugs and 52% of agricultural chemicals contained fluorine [205]. Fluorine is widely used and considered indispensable in molecular drug development. The fluorine atom is similar in size to that of the hydrogen atom, so when exchanged for the ‘–OH’ of the natural siRNA there are no negative impacts on functionality. It is well documented that Fluorine improves cell permeability and provides an improvement in a molecule’s potency [205].

This study reports on the optimization increase RNAi activity gained from 2′-F- modified pyrimidines in dsRNA triggers. We tested three different gene targets (soluble *Trehalase, Cactin* and *Syntaxin 1A*), quantifying mortality after ingestion in individual hemipteran insect vectors, either: (1) the Asian citrus psyllid, *Diaphorina citri*; (2) the Glassy-winged sharpshooter, *Homalodisca vitripennis*; or (3) the Silverleaf whitefly, *Bemisia tabaci*.

Successful RNAi treatments targeting soluble *trehalase* of *D. citri* demonstrated significant reduction of psyllid survival [34,87,106,157]. One report showed that when applied to the soil of potted citrus seedlings hosting psyllid nymphs, this resulted in 100% adult psyllid mortality within days after eclosion [111]. Insect trehalase is evolutionarily conserved having fundamental roles in the chitin production pathway, and in trehalose metabolism, glucose transport and glycolysis in insects [112,155,156,157,158,159,206,207,208]. Thus, effective dsRNA triggers in psyllid can guide the development of effective dsRNA triggers for similar gene targets in insect pests within the Hemiptera (S7 Report, in the Supplemental Materials).

Whiteflies impact food security globally and transmit hundreds of plant viruses on economically important crops including many vegetables such as tomato, melon and other cucurbits, as well as cassava, a staple crop for sub-Saharan Africa. Approximately six species affect cassava production, and one have emerged to accumulate to exceptionally high populations and has become known as the African super-abundant Cassava Whitefly [209]. These whiteflies transmit two important viral diseases in Africa: Cassava mosaic disease (CMD) and Cassava brown streak disease (CBSD), which result in yield losses across Africa of almost 50%, equivalent to >US$ 1 billion annually. In an effort to control *B. tabaci* populations affecting cassava and U.S. vegetable crops, RNA interference (RNAi) strategies were developed.

Our research efforts to develop RNAi to whitefly supports the international Cassava-Whitefly Food Security program and USDA, ARS, whitefly pest management programs (Salinas, CA; Charleston, SC, USA). Since *Syntaxin-1A*, is important in ion channel regulation that is critical for functioning of the insect nervous system, and modulates sexual maturity [180], we selected a 239 nt region that contained a highly conserved region in all four whitefly *Syntaxin-1A* mRNA variants (X1–X4) in the *B. tabaci* species complex. The whitefly *Syntaxin-1A* mRNA transcripts (XM_019056783.1; XM_019056785.1; XM_019056786.1; XM_019056787.1). The conserved sequence was also evaluated when incorporated in a concatamer dsRNA’s that contained trigger sequence(s) to other conserved gene targets to provide suppression across two or more pathways, facilitating increased mortality [210].

Previous reports support that modified dsRNA would increase the mortality of insect vectors (psyllid) upon ingestion [34,87]. Modifications that increase cell uptake, such as increasing fluorine’s, are beneficial for absorption of RNAi biopesticides applied as topical sprays to plant leaves, or as aqueous solutions applied to the soil for plant root absorption [34,69,87,109,110], or in insect baits and diets for insect ingestion. Thus, to optimize absorption and activity we followed the research using 2′-F- modified pyrimidines in siRNA conjugates that are well documented in mammalian cell studies for drug design, that show 2′-F- protects the RNA aptamer sequences from ribonuclease degradation while providing improved biological activity [83,85,189,211,212,213,214,215]. Studies report that one reason the 2′-F modifications are effective is that they provide the best mimic of the natural 2′-OH group being replaced in size and charge, thus being well tolerated while not disrupting the binding affinity of the guide strand to the RNAi machinery. Another reason is that the 2′-modifications usually reinforces the 3′- endo sugar pucker that results in the ‘A-form structure’ in natural RNA. This form increases the binding affinity of the oligonucleotide strand. However, deviations that are too far outside the natural range for structure and charge (≥70%) can result in the modified oligo becoming inactive to RNAi enzymes and RNase H, resulting in loss of RNAi activity [85,189]. The dsRNA triggers in our study incorporated from 35 to 55% 2′-F modifications, well below the 70% limit [189]. For dug development, these benefits in activity led to the use of 2′-F modifications extensively on the primary guide strand [83,189,216,217]. While there are many reports on modification strategies to stabilize aptamers and dsRNA for therapeutics in drug development [83,85,104] there are very few evaluations on modifications specific for increasing activity in insects and arthropods [34,84,86,87,88,91,218]. Since RNAi biopesticides are built on the biological foundation of a natural RNA processing system [73], understanding the molecular mechanism of RNA processing in cells, along with DNA and protein processing, provides new information to develop more innovative treatments that will have greater activity in modulating gene expression [100,219]. Furthermore, breakthroughs in understanding are leading the development of these therapeutics to be safer and more specific with less off-target interactions [49,54,59,62,65,66,104,105,112,138,220,221,222].

The future of RNAi biopesticides will incorporate one or more types of modifications to produce a stable product with consistent activity. The incorporation of a wide variety of modified pyrimidines and/or purines [59,62,84,100], along with modifications in shape designs [218,223] have the potential to produce many different types of effective biopesticides. While 2′-F (fluorine) is just one type of modification that has been widely used, in nature there are a myriad of chemical moieties that encompass ribonucleoside modifications for each nucleoside: adenosine, guanosine, cytidine or uridine [224]. The number of natural modifications researchers have identified include: 111 modifications within transfer RNAs (tRNAs); 33 modifications in ribosomal RNAs (rRNAs); 17 modification in messenger RNAs (mRNAs), and 11 in long noncoding RNAs (lncRNAs) [225,226,227]. Thus, the potential to develop new innovative pest management molecules is enormous. Combine this with the current success of RNA-technologies that produced the COVID vaccines (https://www.nature.com/articles/d41586-020-03626-1, accessed 22 August 2021), and the massive investments made by chemical industries to increase production of chemically modified nucleosides at lower production costs [228], produces a promising future for biopesticides. Biopesticides are an emerging market that can now produce synthesized modified RNA ‘triggers’ at a competitive price approaching that of current commercial chemical insecticides [49,55,57,59,73,84,228,229,230]. Simpler, cost effective approaches have also been reported for producing siRNAs with 2′F- and other natural modifications [52,192,231]. The incorporation of the modified nucleotides into dsRNA triggers, as shown in this study, optimized RNAi activity providing a more consistent result of insect mortality after ingestion by three hemipteran insect vectors. Adoption of modified nucleotides as standard practice would provide an intriguing advantage that could move optimized RNAi biopesticides towards faster regulatory approval and commercialization for agricultural management of insect pests and viral pathogens.

## 9. Materials and Methods

### 9.1. Insect Colonies

Psyllid and whitefly cultures: at the USDA, ARS, insectary, Fort Pierce, FL The colonies of *D. citri*, Asian citrus psyllid, ACP, were cultured at USDA, ARS, Fort Pierce, FL, USA). Psyllid colonies used in were established in 2000 the USDA colony has been reared on *Citrus macrophylla* Wester since 2010 [232,233]. The insectary colony is maintained using procedures similar to those described by Skelley and Hoy (2004) [234], with no infusion of wild types. Colonies were maintained at 26 ± 1 °C, 60–80% relative humidity and a photoperiod of 16:8 (L:D) h. Estimated egg mortality was 3–7%, and nymphal eclosion mortality before adulthood 15–20%. To confirm the absence/presence of the bacterium in the colonies, random subsamples of both plants and insects were tested monthly using a quantitative real-time polymerase chain reaction procedure [15]. We produced four or more biological replicates for independent sample collection. Each treatment group was compared to the TE-Buffer control using Students *t*-test. Whitefly Cultures, reared in the same facilities, USDA, ARS, Fort Pierce, FL, USA, were on tomato, *Lycopersicon esculentum* L. ‘Florida Lanai’, Colonies were maintained at 26 ± 1 °C, 60–80% relative humidity and a photoperiod of 16:8 h (L:D), in walk-in chambers.

### 9.2. Total RNA Extractions

Extractions from untreated and treated samples psyllid, whitefly and plants, were performed using Direct-Zol RNA MiniPrep (Zymo Research, Irvine, CA, USA), following the manufacturer’s instructions. The concentration and quality of RNA were measured by spectrophotometry (NanoDrop™ ND 8000; Thermo Fisher Scientific, Waltham, MA, USA). The cDNA was synthesized from total RNA (1 µg) using the High-Capacity cDNA Reverse Transcription kit (Thermo Fisher Scientific, Waltham, MA, USA). Quantitative PCR assays were conducted using a QuantStudio 6 Flex Real-Time PCR Instrument (Thermo Fisher Scientific, Waltham, MA, USA) and the Syber Green™PCR Master Mix (Thermo Fisher Scientific, Waltham, MA, USA). The data was analyzed using the comparative critical threshold (ΔΔCt) method in which the expression level of the target mRNA dsRNA-treated samples was compared to its expression in untreated samples. Pairs of primers were designed for the target and the reference genes (Supplementary Material) using Primer3 (v.0.4.0 software). PCR efficiencies of target and reference genes were confirmed to be within the range of 90–110% for all qPCR assays. The Shapiro–Wilk normality test and the Levene test of homogeneity of variances were employed to determine the type of distribution for the data obtained in each treatment. T-tests for independent samples or Mann–Whitney U-tests, depending on data distribution, were used to test for significant differences in expression levels (ΔΔCt values) of the target genes between the experimental and control. Internal calibrators may have included *actin, 18s* or *microtubulin* (Supplemental Materials).

### 9.3. qPCR Profile Reverse Transcriptase Real Time PCR

Efficacy of target binding was analyzed by quantitative PCR. Each sample was analyzed in three replicate reactions that consisted of: 2 µL sample, 0.5 µL of 10 µM of each primer, 1 µL ROX reference dye (diluted 1:10), 12.5 µL Platinum^®^ SYBR^®^ Green qPCR SuperMix-UDG and 8.5 µL of nuclease free water. Quantitative PCR was performed in an Applied Biosystems 7500 Real-Time PCR System with the following parameters: 2 min at 50 °C, 10 min at 95 °C, followed by 40 cycles of 15 s at 95 °C and 30 s at 60 °C. Melting curve analysis was also obtained following completion of the final cycle. Primers used for studying expressions genes with reference to psyllids included *actin,* elongation factor *1-alpha (EF1-a), wingless (Wg)* and ß-tubulin (Supplementary Material). After an initial activation step at 95 °C for 5 min, 40 cycles (95 °C for 30 s, 57 °C for 30 s, 72 °C for 30 s) were performed. Cycle threshold (Ct) values were determined using the 7500 Fast software supplied with the instrument. Levels of transcript expression were determined via the 2–ΔΔCt method [200] by normalizing the amount of target transcript to the amount of the internal reference transcripts mentioned above.

### 9.4. Pre-Trial Screening to Evaluating Activity of Three Trehalase-dsRNA Triggers Targeting Different Regions within the Same mRNA Target, Soluble Trehalase in the Asian Citrus Psyllid

To select the most active dsRNA trigger to an mRNA target, a pre-screening replicated trials of dsRNA triggers were conducted (Figure 2). Three regions of the *Trehalase* mRNA were selected for dsRNA production (Supplemental Materials). The dsRNA trigger that induced the strongest RNAi response on the *Trehalase* mRNA was advanced for use in additional *inPlanta* feeding bioassays [108]. This was the normal pre-screening trial for dsRNA’s prior to conducting larger feeding bioassays.

For each of the three trials, two plants were prepared for each treatment, in a manner that would produce new growths called ‘Flush’ that would be cut and used for feeding assays. Preparation was carried out 4 wk prior by removal of all leaves and letting the citrus seedling produce new growth flush. The trimmed seedlings were kept under 16:8 L:D photoperiod with additional LED lighting. The seedlings having suitable new growth were then randomly separated into groups. Two potted citrus seedlings for each treatment were then placed into a screen tent cage (BugDorm^®^) to which 300 adult psyllids were added being left for 48 h for females to oviposit eggs on the new growth. Then the adults were shaken from the plants, and any remaining adults removed using an aspirator. The individual citrus seedlings were then placed into freshly set up screen tent cages for each trial.

Analyses of 4th instar nymph used citrus cuttings of flush placed into plastic centrifuge vials (2.0 mL). Each treatment group had six to eight cages, depending upon the number of new growth flush. For adult feeding bioassays with cutting there were eight cages with flush, which received 15 adult psyllids each as biological replicates to quantify RNAi mortality effects. A second set of four cages were set up for sampling of individual adult psyllids over time (3 d, 6 d, 8 d and 10 d) for expression analyses. The *D. citri,* psyllid colony was maintained at the Insectary, USDA, ARS, Fort Pierce, FL. Colony gender ratio was previously determined to be 1:1 the week prior to use in experiments. Collected psyllid samples were homogenized in RNA-Later, and frozen at −80 °C until analyzed with qPCR.

### 9.5. Comparison of Trehalase-dsRNA with an Insecticidal Soap Applied as Exogenously Sprays

The topically applied exogenous trehalase–dsRNA (70 µg per plant) was compared to a water control, and a 5% Insecticidal Soap, (Safer^®^Brand Insecticidal Soap Concentrate diluted to 5% in water (Woodstream Corporation, Lititz, PA, USA) (Figure 3). The soap is marketed as being composed of approximately 50% potassium salts of fatty acids. The Safer Soap was reported to be lethal to nymphs and adults when sprayed directly onto psyllids at a concentration of 2% *v*/*v* in water [199]. In this case, 16 potted *Citrus macrophylla* seedlings, which had been cut back, removing all petioles and leaves, to expose a seedling trunk of 20 to 28 cm in height, these were kept under a 16:8 light: dark cycle (7000 Lumens, 4000K Cool White LED light, Model #SHOP/4X4/840/HD) to induce new growth, ‘flush’. When each seedling had regrown having an average of six new sprouted petiole growths (each about 2.5 to 4 cm in length) the seedlings were sorted into four groups of four plants per treatment. The stock dsRNA was prepared with approximately 280 µg of trehalase-dsRNA diluted in 40 mL of filtered water (Whatman^®^ water system, Thermo Fisher Scientific, Waltham, MA, USA). From this solution, aliquots of 5 mL were loaded into an 8 mL glass vial fitted onto a Preval^®^ Aerosol sprayer (Coal City, IL, USA) and applied to citrus seedlings. Potted citrus seedlings were grown in a glasshouse with additional lighting to produce 16:8 L:D, photoperiod to produce new growth shoots, called ‘flush’. Before exposure to insects the citrus seedlings with the new growth ‘flush’, were sprayed with 5 mL of treatment solution (either water alone, or one of the three *trehalase*-dsRNA) making sure to cover the top and underside of the foliage surfaces of each seedling. After the first spray application each treated, seedling was allowed to adsorb the solution for 5 h. Then a second application of the same solution (5 mL) was applied to each seedling, thus each seedling received a total of 70 µg dsRNA. All treated citrus seedlings were allowed to adsorb and dry for 24 h. Each treatment group of four citrus seedlings were then placed into an insect cage and 300 adults *D. citri* were released into each cage. Plants were maintained at 25 ± 2 °C and 60 ± 5% RH in an insectary under 16:8 h light: dark ambient photoperiod. Plants were watered as needed. The seedlings remained in the cages with psyllids for three days, after which the adult psyllids were removed using an aspirator. The adult psyllids used for oviposition were collected from breeding colonies of psyllids with a gender ratio of 1:1 and which were between 10 to 15 days old. Adult females can modulate the number of eggs they lay per day dependent upon host quality and presence of other psyllid eggs. In the plant-feeding Hemiptera, psyllid [235] and whitefly [236] the life cycle includes an egg stage and five instar molts to become an adult. The first instar nymphs are docile and move only when disturbed or over-crowded. About 4 days after the eggs hatch the nymphs move to or remain on the new growth tender shoots to feed. The nymph stage lasts about 12 days. Development from egg to adult varies, in psyllids it takes from 14 d at 28 °C, to 49 d at 15 °C [235]. New adults reach reproductive maturity within 2 to 3 days, oviposition begins about 2 days after mating and adults can live for several months. The psyllid reproduces best at temperatures between 25 and 28 °C. Under these conditions, the female lays between 400 and 800 eggs and remains alive for about 50 days [237]. The 6 flushes per citrus seedling tree averaged 25 to 60 eggs per flush. Egg counts were made using a stereomicroscope. The plants were gently shaken to remove most of the adults, then they were moved into a light box to remove the remaining adults using an aspirator. After 4 hr all plants were rinsed with tap water. Then placed inside a fresh ‘bugdorm 2120′ insect screened tent cage for the duration of each trial 24 d (BioQuip Products Inc., Rancho Dominguez, CA, USA). The psyllid developmental time in these trials from egg to adult took from 21–25 d (21–25 °C). Thus, we selected 24 d for the length of trials so surviving nymphs had time to eclose as adults. Analyses of oviposition between treatment groups with one-way ANOVA (Table S2) in the Supplementary Material.

### 9.6. RNAi Feeding Bioassay Whitefly on Okra Plants

Whiteflies, *B. tabaci,* were given an 8-day feeding access period on okra seedlings treated with unmodified, canonical Syntaxin-dsRNA versus modified pyrimidines Syntaxin-dsRNA (2′F- C, 2′F-U). Each dsRNA was applied as an aqueous soil treatment to potted okra plants.

### 9.7. Whitefly RNA Extraction

*Bemisia tabaci* adults were obtained from the USDA-ARS insectary, and reared on cherry tomato (Ft. Pierce, FL, USA) and homogenized in TriReagent^®^ (MRC, Cat. No. TR118, Thermo Fisher Scientific, Waltham, MA, USA) using a plastic pestle. Total RNA was extracted from the homogenate using a Direct-Zol™ RNA MicroPrep kit (Zymo Research, Cat. No. R2062, Irvine, CA, USA) and quantified using a NanoDrop™ND-8000 spectrophotometer (NanoDrop Technologies Inc., Wilmington, DE, USA).

### 9.8. Construct Design

The complete cds sequence for *Syntaxin*-1A in *B. tabaci* (KC161217.1) was translated to a protein sequence on the ExPASy Bioinformatics Research Portal (http://www.expasy.org, accessed 8 July 2021) [238]. The region of the amino acid sequence that included the SNARE complex was identified and used to design a custom 160 bp construct identified in the mRNA labeled as WH9 [210]. The Primers to sequences were designed using Primer3web (v4.1.0) to amplify this construct through RT-PCR in (Table S1) in the Supplemental Materials).

### 9.9. RT-PCR

A One-Step RT-PCR reaction was performed to validate the *Syntaxin*-1A gene in *B. tabaci* using the Invitrogen SuperScript™ One-Step RT-PCR with Platinum^®^ Taq (Cat. No. 10928-034) (Thermo Fisher Scientific, Waltham, MA, USA). Each reaction consisted of 100 ng total RNA, 1.0 µL of 10 µM of forward and reverse primer, 25 µL of 2X reaction mix, 1 µL RT/Platinum^®^ Taq and sufficient nuclease free water to bring final volume of each reaction to 50 µL. PCR was performed in an MJ Research Peltier Thermalcycler™ (PTC-200) using the following parameters: 30 min at 50 °C, 2 min at 94 °C, followed by 39 cycles of 15 s at 94 °C, 30 s at 60 ° and 30 s at 72 °C, and a single final cycle of 72 °C for 10 min. The reaction was fractionated by electrophoresis for 35 min in a 2% agarose gel stained with ethidium bromide. All reactions generated an amplicon that was the appropriate product length. The bands were excised from the gel and purified using the Macherey-Nagel NucleoSpin^®^ Gel and PCR Clean-up kit (REF 740609.250) (Thermo Fisher Scientific, Waltham, MA, USA).

### 9.10. T7 Template Synthesis

The *Syntaxin-1A* dsRNA construct primers were synthesized with a 5′ T7 promoter sequence (5′-TAATACGACTCACTATAGGGAGA-3′) and used to generate T7 template for dsRNA synthesis. A conventional PCR reaction was performed to incorporate the T7 sequence using the Invitrogen Platinum^®^ PCR SuperMix (Cat. No. 11306-016). Each reaction consisted of 100 ng purified RT template, 1.0 µL of 10 µM of forward and reverse T7 primers, and 45 µL Platinum^®^ PCR SuperMix (Invitrogen, Thermo Fisher Scientific, Waltham, MA, USA). PCR was performed in an MJ Research Peltier Thermalcycler (PTC-200) using the following parameters: 3 min at 94 °C, followed by 39 cycles of 15 s at 94 °C, 30 s at 60 ° and 30s at 72 °C, and a single final cycle of 72 °C for 10 min. The reaction was fractionated by electrophoresis for 45 min in a 2% agarose gel stained with ethidium bromide. All reactions generated an amplicon that was the appropriate product length. The bands were excised from the gel and purified using the Macherey-Nagel NucleoSpin^®^ Gel and PCR Clean-up kit (REF 740609.250) (Takara Bio USA, Inc., San Jose, CA, USA).

### 9.11. Synthesis of dsRNA and siRNA Production

Unmodified, canonical *Syntaxin-1A*-dsRNA was synthesized using the Ambion^®^ MEGAscript^®^ RNAi Kit (Ref. No. AM1626) (Thermo Fisher Scientific, Waltham, MA, USA). The modified, noncanonical, 2′-F-cytosine and 2′-F-uracil dsRNA was synthesized using the Lucigen^®^ DuraScribe^®^ T7 Transcription Kit (Cat. No. DS010925), per manual instructions (Lucigen Corporation, Middleton, WI, USA).

The synthesis of dsRNAs was performed using the MEGA-script™ RNAi kit (Thermo-Fisher Scientific, Waltham, MA, USA) according to the manufacturer’s instructions. The template DNA for dsRNA production was generated by PCR amplification using insect specific primers containing T7 promoter sequences tailed at the 5′ end of each primer (Table S1). Chemically synthesized dsRNA’s were purchased as manufactured from (Genolution™ Inc., Seoul, Republic of Korea) for experimental dsRNA negative controls were either Chinese Sacbrood Virus capsid, CSBV-dsRNA, sequence product size of 114bp or a dsRNA tor Green fluorescent protein (dsGFP, AJ306911.1), product size 480bp. After synthesis, dsRNA was purified using the filter cartridge provided in the kit. The dsRNA concentrations were measured using a NanoDrop ND8000 spectrophotometer (ThermoFisher Scientific, Waltham, MA, USA).

### 9.12. RNAi Suppression of Psyllids Feeding on Citrus Seedling Trees

Exogenous, foliar application of dsRNA and soil applied aqueous dsRNA solution methods as in Ghosh et al. (2018) [109] and Hunter et al. (2020) [87]. The feeding bioassay-methods described using citrus cuttings followed the *inPlanta* System, iPS, Andrade and Hunter (2017) [108].

### 9.13. Whitefly Feeding Bioassay

A bioassay was performed to compare the efficacy of unmodified, canonical dsRNA vs modified, non-canonical dsRNA for improving RNAi induction and pest suppression. Okra seedlings approximately 2 inches in height with two true leaves were transplanted into 50 mL conical tubes. The soil was allowed to dry for approximately 72 h. The dsRNA (40 µg) was suspended in 10 mL of water. The soil volume was equivalent to a 50 mL conical tube and was allowed to moderately dry prior to soil drenching with the dsRNA aqueous solution. Seedlings were allowed to absorb dsRNA for 48 h. Approximately 25 whiteflies were then added to each seedling covered with a ventilated bioassay tube. Experiment was allowed to run for 8 days at ambient room temperature, with a 16:8 light period. Three whiteflies were collected from each tube on day 7 and processed for RNA to quantify gene expression through RT-qPCR.

### 9.14. Gene Expression Analysis

Three *B. tabaci* adults were collected on day seven from each treatment group and the total RNA was isolated from each individual using the Direct-Zol RNA MicroPrep Kit (Zymo Research, Cat. No. R2061, Irvine, CA, USA). The purity and concentration of RNA was determined using a Nanodrop ND-8000 spectrophotometer (NanoDrop™ Technologies Inc., Wilmington, DE, USA). Gene-specific real time PCR primers for *Syntaxin-1A* mRNA were designed using Primer3web (v4.1.0) and were forward: 5′-CAAGGAAATTCTGCTGTGTTCA-3′ and reverse 5′-ATATCCGCATGTCTTGCTT CA-3′. The elongation factor *1*-α gene was used as an endogenous control: forward: 5′- TAGCCTTGTGCCAATTTCCG-3′ and reverse: 5′-CCTTCAGCATTACCGTCC-3′. qRT-PCR was performed on an ABI 7500 Real Time PCR (Applied Biosystems, Carlsbad, CA, USA) system using the Invitrogen SuperScript III Platinum SYBR Green One-Step qRT-PCR kit (Cat. No. 11736-059) (Thermo Fisher Scientific, Waltham, MA, USA) with 10 ng of RNA per reaction. Relative gene expression was calculated based on three biological replicates, each with three technical replicates.

### 9.15. Statistical Analysis

The data were summarized as the mean ± SE (standard error) for all data sets. The data were then subjected to a One-way analysis of variance (ANOVA) [239] using Analyse-it^®^ Statistical analysis add-in for Microsoft Excel, significance, Excel (version 7.2.10 68) and/or ANOVA using Social Science Statistics [240], (https://www.socscistatistics.com/, accessed 18 August 2021). Means separation post-hoc test Tukey (HSD) with differences considered statistically significant at the 5% level (*p* ≤ 0.05). The calculated Eta Square, Eta^2^ is calculated the same way as the R Squared, is the correlation ratio [239]. The Eta^2^ is used as a measure of strength of association based on the Sum of Squares (SS) in the context of analysis of variance (https://www.statisticshowto.com/eta-squared/, accessed 18 August 2021). The Eta square is computed as the division between group Sum of Squares (Between Group SS) and the Total Sum of Squares values (Total SS) in the ANOVA output table. Thus, the “SSeffect is the sums of squares for the effect you are studying. The total SS is the total sums of squares for all effects, errors and interactions in the ANOVA study. Alternately this may be written in the formula: Eta^2^ = SSbetween / SStotal. Generally, the Eta square value is listed after the p-value. The correlation ratio cannot prove causal direction such as other types of correlations and associations, however, Eta^2^ can measure the level of causal direction and for this reason the correlation ratio only varies from zero to one (https://www.statisticssolutions.com/free-resources/directory-of-statistical-analyses/correlation-ratio/, accessed 18 August 2021). Experiments were performed in triplicates. All samples were three or more biological replicates, with three or four technical replicates. Expression analyses included for each biological replicate qPCR was performed with a minimum of three technical replicates.

## Data Availability

All datasets’ links to data generated or used for this study are either included in the article or through the web links provided in the Supplemental Materials [see S7. Gene Data-mining from De Novo Genomes (Psyllid, Whitefly, Leafhopper)], and through NCBI using listed accession numbers provided.

## Supplementary Materials

The following are available online at https://www.mdpi.com/article/10.3390/plants10091782/s1, Figure S1: Global Distribution Maps. CABI-Hemipteran Vectors; Figure S2: The three regions of *Soluble Trehalase-1, -2, -3-* as *dsRNA* tested for activity; Figure S3: Phylogeny of the *Syntaxin 1A* mRNA from *Diaphorina citri*, Asian citrus Psyllid; Figure S4: Phylogeny of the *Cactin* mRNA sequence, from *Diaphorina citri*, Asian citrus Psyllid; Figure S5: Modification in oligonucleotides and their properties; Table S1: Primers used in this study.; Table S2: Mean Number of Psyllid Eggs, per Six Petiole ‘Flush’ per plant; S7 Report: Gene Data-mining from De Novo Genomes (Psyllid, Whitefly, Leafhopper).
