# Supplementary material for: Optimizing Efficient RNAi-Mediated Control of Hemipteran Pests (Psyllids, Leafhoppers, Whitefly): Modified Pyrimidines in dsRNA Triggers
Source: Plants (Basel). 2021 Aug 26;10(9):1782. doi: 10.3390/plants10091782 (PMC8472347; doi:10.3390/plants10091782)
Supplement: Supplementary file 1 [file plants-10-01782-s001.zip › plants-1322767-supplementary/plants-1322767-Supplemental Files Hunter/Supplemental TABLE_S1_HUNTER- Primers.pdf]

Optimizing Efficient RNAi-mediated Control of Hemipteran Pests (Psyllids and Whitefly): Modified pyrimidines in dsRNA Triggers.

Wayne Brian Hunter[1]* and William M. Wintermantel[2]

Supplemental Materials_S1. TABLE

**Table S1. Psyllid- Primers used for dsRNA production, RT-qPCR, and siRNA sequences**		
**Name**	**Sequence**	**Position**
dsAK-F	**TAATACGACTCACTATAGGGAG**TGGCATTCTTGTATGGCGTA	57
dsAK-R	**TAATACGACTCACTATAGGGAG**GCCTGCAAGAATCTGTCTCC	730
dsAK 5′-F	**TAATACGACTCACTATAGGGAG**TGGCATTCTTGTATGGCGTA	57
dsAK 5′-R	**TAATACGACTCACTATAGGGAG**TGAAGCCCTTGTGGTAGTC	420
dsAK 3′-F	**TAATACGACTCACTATAGGGAG**ACCCGGACTCTGGAGTAGG	319
dsAK 3′-R	**TAATACGACTCACTATAGGGAG**GCCTGCAAGAATCTGTCTCC	730
Cactin	***Diaphorina citri* (XM_008474513.2)** *Note:* had a 400bp deletion.	308 bp region
Cactin	**MCOT for *cactin* (MCOT20318.0.CT)*Better for dsRNA synthesis**	300 bp region
Cac-F	GGAAACCGCGATACTTTAACA	
Cac-R	GGAACTGACATCTGAAGCCTCT	
**rtPCR**		
Cac-F	AGCGATGAAACCTCTGCTACA	
Cac-R	TCAACGGGTTACTTTCTGGTG	
	**Trehalase**	
Treh-F	AGTATACGGGCGACACCAACTTTAT	
Treh-R	AACTGCTTGAAGCCATTCATGAG	
	**>MCOT10768.0.MT *Diaphorina citri* Syntaxin 1A**	
Sx-F	GATACCGGGAACCCAGCAG	
Sx-R	GTTTCGAGTCCGCGGATCT	
	**Elongation factor 1-α gene**	
Elf-F	ACCACCACCAACACATCTAC	
Elf-R	ACTTCTTCTCCTCCTCTCATCT	
	**Arginine Kinase**	
AK_quant-F	CGGACTTGAGGGAGAACTGA	611
AK_quant-R	GTGGTAGATACCGCGACCAG	776
	**Tubulin**	
α-Tub-F	GCGTCTCTTCGGTTTGACGG	896
α-Tub-R	CACTTCACCATCTGGTTGGC	1092
**T7**	5′-TAATACGACTCACTATAGGGAGA-3′	
**Chinese Sacbrood Virus Capsid(CSBV-dsRNA) Control**		
CSBV-F	TTCACAACAGGGAGCTTGTG	Product size-114 bp
CSBV-R	CCCCATAACCTTCTCCACAG	

**Green Fluorescent Protein – (GFP-dsRNA) dsRNA Control**		
GFP-F	GGTAAAAGGACAGGGCCATC	39
GFP-R	TCAAGGAGGACGGAAACATC	237
dsGFP-F	**TAATACGACTCACTATAGGGAG**CCAACACTTGTCACTACTTTCTCTT	1
dsGFP-R	**TAATACGACTCACTATAGGGAG**GTAATGGTTGTCTGGTAAAAGGA	480
	Green fluorescent protein (dsGFP, AJ306911.1), product size 480bp.	
**Leafhopper- Primers used for dsRNA production, RT-qPCR, and siRNA sequences**		
Cactin	*Homalodisca vitripennis* (HVIT015866-RA)	
Cac-F	CGGGATTCGAGTGGAATAAAT	
Cac-R	CTATGGTTCCACTTCAAGCGA	
	**Elongation factor 1-α gene**	
Elf-F	ACCACCACCAACACATCTAC	
Elf-R	TCCTCCGTAGTGACCAGTGTC	
**Whitefly - Primers used for dsRNA production, RT-qPCR, and siRNA sequences**		
	**PCR primers for *Syntaxin-1A* mRNA**	
Sx-F	CGAGAGTTGCATGACATGTTT	
Sx-R	AAATATCAGAGTAAAGCGCGG	
	**Elongation factor 1-α gene**	
Elf-F	TAGCCTTGTGCCAATTTCCG	
Elf-R	CCTTCAGCATTACCGTCC	
	National Center for Biotechnology Information, NCBI	
	https://blast.ncbi.nlm.nih.gov/	

T7 promoter primer region in bold.

Chinese Sacbrood Virus(CSBV-dsRNA): Production from Genolution, company (AgroRNA):

GATTTGGCTATTTGGGTCCTGTCTCCATCATTTGGAATGTTTAAGGATATAAGGAAGTTTATAGCTACAG
ATGAGGACCTATCTAAACCAATTACTACGGAAGGGTCCTTATTATTGGCCCCAACTAATCGTAACCCAGT
GCTTAAGGAACAGAGTATAGAGATACTGGGCCTACAAAATGAAATGCAAGTATCTGAGTTAAATGGCACA
GTATTCTATGCGAGTGATGTAATTTGTTATGATTATTCACAACAGGGAGCTTGTGGATCTCTGTGCTTCT
TGTCCCGCTCCCAAAGACCTATTGTGGGAATGCATTTTGCTGGTCGAGGTGAGGGGTCCTGTGGAGAAGG
TTATGGGGTTATTTTAACTAAAGAGGCTATTGGGGATATTTTAGCATTGAAGTCTCAACCTGTGGTACAG
TTGGAAGATTGGGAAGGACCCAGTTTAGAA

**OLIGO** Primer	start	len	tm	**gc%**	any	3' seq
**LEFT PRIMER**	250	20	59.87	50.00	5.00	**TTCACAACAGGGAGCTTGTG**
**RIGHT PRIMER**	363	20	59.40	55.00	2.00	**CCCCATAACCTTCTCCACAG**

PRODUCT SIZE: 114 bp

Arginine Kinase *Diaphorina citri*, Production from Genolution, company (AgroRNA):

GGCATTCTTGTATGGCGTACGTGAAAAATTATTGGAGAATGTGAAGGAGTCAATGCAACCGGGAAATGAA
ACTGGAAGTACAAAAATGGTTGACCAAGCAACTCTTGACAAGCTAGAAGCTGGCTTCGCCAAGTTCCAAG
CCACAGAAAGCAAGTCCCTGCTCAAGAAATATCTGACCAAAGAAATTTTCGATGCCCTCAAAGTGAAGAA
AACCTCGTTCGGATCTTCCCTGCTGGATGTCGTCCAATCAGGTTTCGAGAACCCGGACTCTGGAGTAGGT
ATCTACGCTCCCGACGCTGAGGCTTACATCGTGTTTGCTGACCTGTTTGACCCCATCATCGAGGACTACC
ACAAGGGCTTCAAAAAGACTGACAAGCATCCCCCCAAGAACTGGGGCGATGTC

OLIGO	start	len	tm	gc%	any	3'	seq
**LEFT PRIMER**	182	20	60.02	40.00	7.00	3.00	**AATTTTCGATGCCCTCAAAG**
**RIGHT PRIMER**	326	20	59.75	50.00	2.00	2.00	**ACAGGTCAGCAAACACGATG**

PRODUCT SIZE: 145 bp

>MCOT10768.0.MT. Predicted. *Diaphorina citri*, Syntaxin-1A. Blue Region dsRNA. Data: www.citrusgreening.org

CTTTCGGAAATCAGTCGAATTTTTTCTTCTTCTCTTTTTCGGAACTTTTTCGTCTGAAAAGGAAAAGGGTAGTTTTTGGGCTTTTC
GGGGGGCGCAGTCGCCGTTTAGAAGACGGCATTGCAGAAATTTATTGATTTTTATGTCATTCTAGTTAAGTTCAGTGTATTCTCTA
GTGTAGTTTATATTATTAGCTTTTAAAAAAAATTCCGTATAAAAATTGTGTTATTAAAATAAATCGTTAATGGGACTCCCGTGTGA
TTTAAGTTTAAAGTGTTTCTTTTAGTATTTAATTAAAGTGTTCGGTTAGAAACATGACGAAGGATAGGCTAGCCGCTTTAAAAGCT
GCTCAGAGTGATGATGATGATGTGGGACCGGAGGAGGTGGCGGTCAATGTGGACGGGAGAGATGGATTCATGGATGAGTTCTTTGC
TGAGGTGGAAGAAATTCGGGAGATGATAGACAGGATACAGACAAACGTAGAGGAAGTCAAAAAACGACACAGTGCCATTTTGTCCG
CGCCTCAGACCGATGAAAAAGTAAAACAAGAACTAGAAGACTTGATGGCAGACATTAAAAAAACAGCCAACAAAGTTCGTGCCAAA
TTAAAAGTGATCGAGCAGTCTATCGAGCAAGAGGAGCAGTCGAACAAATCGTCGGCAGATCTGAGGATACGCAAGACCCAACACTC
CACCCTGTCCAGGAAGTTTGTCGAAGTGATGACGGAATACAACCGGACGCAGACCGACTATCGCGAGCGATGTAAAGGCCGGATAC
AGCGACAGTTGGAAATCACGGGTAGAACAACAACAAATGAAGAACTAGAAGCAATGTTG**GATACCGGGAACCCAGCAGTGTTCACA**
CAAGGTATAATCATGGAGACGCAGCAGGCCAAGCAGACCCTGGCGGACATAGAGGCCCGGCACGCGGACATCATCAAGCTGGAGAA
CTCCATCCGAGAACTGCACGATATGTTCATGGACATGGCCATGCTGGTCGAAAATCAAGGTGAGCTTGTAGACAATATAGAGCATC
ACGTCCAGCAATCTCAGAACCAAGTGCTCGTGGCCAAAGAAGAACTAGTTATAGCGGAAGAATATCAAACCAAAGCCAGGAAGAAA
AAGCTGTTCATGCTCATCTGTCTGTTCGTCCTGTTCATCCTGCTGGGGGCGTTCCTGTTGTCGTTCATCCAATGATGTTTCGAGTC
CGCGGATCTCGATGTCAGCGAAGACGAGGCGGAGAACTTCGAAGCCACGTATAAACAATTCCTATAAGACGAGTTCACGTATAAAC
AATTCCTATAAGACGAGTTCACGTATAAACAATTCCTATAAG

>KC161217.1 *Bemisia tabaci* Syntaxin-1A mRNA, complete cds.
Length: 882. **Blue Region dsRNA.**

ATGACGAAGGATAGATTGGCTGCTCTGAAAGCGGCCCAAAGTGATGACGATGAAGCGGGCTCCGATGATGTGGCCGT
CAACGTGGAAGGAAAGGATGGTTTCATGGATGAATTTTTTGCCGAGGTTGAAGAGATACGAGAGATGATAGATCTTA
TACAAAGGAATGTAGAAGAAGTGAAGAAAAAACATAGTGCCATCCTTTCAGCTCCTCAGACCGATGAAAAGGTCAAG
CAGGAGCTGGAGGACCTCATGGCCGACATTAAAAAAACTGCCAACAAAGTTCGTGCCAAATTAAAAGTGATTGAACA
GAATATAGAGCAAGAAGAACATACAAATAAATCGTCTGCAGATTTAAGGATACGCAAAACCCAACACTCCACGTTAT
CAAGAAAGTTTGTTGAAGTCATGACTGAATATAACCGAACACAAACCGATTATCGTGAAAGGTGCAAAGGAAGAATA
CAAAGGCAATTAGAAATCACTGGAAGAACAACGACAAATGAGGAATTAGAAGAAATGTTAGAACAAGGAAATTCTGC
TGTGTTCACTCAAGGGATAATAATGGAAACACAGCAGGCAAAACAAACTCTAGCAGATATTGAAGCAAGACATGCGG
ATATTATAAAATTAGAAAATTCTATACGAGAGTTGCATGACATGTTTATGGATATGGCTATGCTTGTTGAAAACCAG
GGAGAAATGATTGACCGTATCGAATATCATGTAGAACATGCGGTCGATTATGTTCAAACTGCAACACAAGATACTAA
GAAAGCATTAAAATATCAGAGTAAAGCGCGGCGGAAAAAGATCCTGATCATTATCTGCTTGATAATTTTTCTCATCG
TTTTAGTTGTCATTTTCGCCACATTTTTTGGATGA

Figure_*Syntaxin-1A*-dsRNA. *Bemisia tabaci.* T7 promoter primer region in **bold**.

TAATACGACTCACTATAGGGAGA*CGAGAGTTGCATGACATGTTT*ATGGATATGGCTATGCTTGTTGAAAACCAGGGA
GAAATGATTGACCGTATCGAATATCATGTAGAACATGCGGTCGATTATGTTCAAACTGCAACACAAGATACTAAGAA
AGCATTA*AAATATCAGAGTAAAGCGCGG***TCTCCCTATAGTGAGTCGTATTA**

>XM_008474513.3:192-2024 PREDICTED: *Diaphorina citri* Cactin (LOC103509881), mRNA. NCBI.

ATGGGTAGAAGTGAGCATTCCTACAGATCTTCAGATCACAGTTCAAAACACAAGGAGAGCAAGTCAAGCTCACGAGA
TCATCATTCTAGTTCTGATCGGTACCACCAAGATGAGCGGAGTAAATATACTGAATCTTCCTCATCACATAGAAGGC
ATGATGCATTGTCAAGGTATGATGAAGAGCCTAGTAGAAAACGTGAAGAATATAGAAGACATGAGGAACAGAAATCA
AGAGAGGATAAAACTTCCAGGGCATCCAGTAGTCACCAGTCAAGGAAAAAGTCACACTCATCTTCAAGAAAACATTC
AAAGAGTTCCAAGCATAGACACTCCTCCTCTTCCTCCTCATCATCTTCATCAGAAAGTTCTGCAGATTCTGAGAAAG
AGCTCAGACTCTTGAAACAACTGGCTGAGGAGAGGGAACGGAAAGTAAGAGAGAAGAAGAAAATGAAGGAGTTGGTG
AAAGCTACAGAGACCCCTGAGGAAAAACGCCTACGAAGGTTACTTAAAAAGGAAGCCAAAGAAAGGAAGCGAAAGGA
ACAGATGGGCTGGGACAATGACTACATGCATTATACCAACTCGGATAACCCTTTCGGTGATAGCAATCTCCTCACAA
ACTTTGTATGGAGTAAGAAGTTAGACAAGGAGGGACTGACAGGAGTGAGTGAGACAGAGCTACAGGAACAGAACCGG
AGGAAGATGGAGGAGAATAAGAGAGAGTTGGAGAAGGTAAAGAAGCGTCGTCAAGAGAGAGAAGCGGAGAGGGCAGA
GAGAGAGGAAGAGATGGCGTTAACTCAGCGTGGCAAGGAGGCAGCTCAGTTTGAAGAGTGGCAGAGACAGGAAGATC
AGTTCCATTTGGAACAGGCTAGGCTGAGGAGTACAATACGTATACAAGAAGGACGGGCCAAACCTATCGACCTCCTC
GCCAAATACATCGGGTCCGAGGCAGAAGTGGATGCTGTGGAGATGCACGAACCATACACATACCTTACCGGTCTGGC
TATTAAAGATCTGGAAGACCTACTGGAGGATATCAAAGTATACATGGAACTGGAAAAGGGAGAGAATGAGGCGTATT
GGAATGATATCAGTATTATTGTAGAGGATGAACTGCATGGATTGAGGAAACTGGAGAAACAAGGACACAGCGACCTG
ACTAGACGGGAAGGTATTCACGAGTCTGTAGCACAAGATGTGGCCTCTGTATTCAAAGGTAAAACTACAGCACAATT
AGAAGCTCTACAGACACAGATCGAACTGAAAATATCCAGCAAGACAGAGAACATAGATGTGGGATACTGGGAAACTT
TACTATCACAACTTAAAGGTAATTTCGTGTTCCTGAACGTGATCACAGCTGAAGAGAAGGCACTACAACGTGAAGCA
AGGAAAGGTATGGGCGGTGATGAGGCTGAGTTCAGTGTGGAGAGTTTACTTGATCCGCAAGTATACCTGTGGTCCGA
TAAATATCGCCCTAGGAAACCGCGATACTTTAACAGAGTTCACACTGGTTTCGAGTGGAACAAATACAATCAAACCC
ATTACGATATGGACAATCCTCCTCCCAAGATAGTACAAGGCTACAAGTTTAACATATTCTATCCTGACTTGATTGAT
AAGAACTCTACACCTCAATACTTCCTGAATACATGTGCTGATAATCAGGACTTTGCTATTCTGAGATTCCACGCTGG
TCCTCCATATGAAGACATTGCATTCAAGATAGTCAACAGAGAATGGGAGTACTCCTACAAGAGAGGCTTCAGATGTC
AGTTCCATAATAATATATTTCAGTTGTGGTTCCATTTCAAGCGATATCGATACAGAAGATAG

>gnl|*Homalodisca vitripennis* transcript v0.5.3|HVIT015866-RA, **Putative Cactin mRNA. Blue Region dsRNA**

CTATGTACTGTGCTGTGTTGTCAGGGTGGATGAGGATCAAGTGGCCAACGCAATGCTGACAGAGAGTTTC
GCCGACTACGACTCAGGACAGTACAGTCCCCGGTACCTGAGCCAGTCACAACTAGAACCAGGGACACTGG
TCACTACGGAGGAGGATGATGCCAAGAGGTTGGTGTTTGCCAGACTACAGGTGCAGGGCACTGGCAAGAG
TGTGCAGCCAGCCACGTCTCTGGAGGAAGAGGTGCTGCAGAGAGAAGCTCGCAAGGGGATGACCAACGAT
GAAGCAGAATTCTCTGTAGAGTCTATCCTGGACAGTCAGGTCTATCTGTGGTCTGACAAGTACCGTCCTC
GCAAACCAAGATACTTCAACAGAGTTCACA**CGGGATTCGAGTGGAATAAATACAACCAGACTCACTACGA
CATGGACAACCCTCCACCCAAGATTGTGCAGGGCTACAAGTTCAATATCTTCTACCCTGACCTCATCGAC
AAGAATGCAACTCCAGAGTACTTCTTGACACCATGTCCGGAAAATCATGATTTTGCAATCCTCCGGTTCC
ACGCAGGACCACCGTATGAAGACATAGCCTTCAAGATTGTGAACAGAGAGTGGGAGTACTCGTATAAACG
TGGCTTCCGCTGCCAGTTCCACAACAACATCTTTCAAGCGA**TATCGCTACAGGAGATAAAGTTCCGGTTG
AACATTCGACCTGTGTATCTGTGTAAATAAAATTGTATATCATTTTTAAA

>XM 019060862.1:186-2267 PREDICTED: *Bemisia tabaci* Cactin (LOC109043602), mRNA. NCBI.

ATGCATCGCTCACGGTCACATCATCGTAATAGACACAGGGATGAATCATCATCGAGGTCTTCACGGAAGTCAAAAAG
CCACAAGCGGGACAAGAAATCCAAGAGCTCCAGGCGAGATAGAAAATCTCGACGAAGGCACTCATCAGAGTCATCAT
CTCGTCCTTCATCCTCCTCCTCATCCACATCCTCCTCTTCCTCTGCGTCTTCCTCATCTTCTTCATCAAGTGCCTCA
GATAAGGAAACGAAACTCCTGATAAGATTGCAAGAGAAGCGGAAAAGACAGCAGGAAGAAAGGCGTAAACTGAAAGA
GCTCCAGAAGGCCCAAGAAACCCCCAAAGAAAAGAGAATCAGACGACTGCTGAAGAAAGAGGCCAAAGAGAAAAAGA
GGAAACAACAAATGGGATGGGACAATGATTACCTACACTACACAAATGTGGACAATCCTTTTGGGGATAATGATCTT
CTTTCAACATTTAGATGGGATAAAAAGCTGGAGAAAGAAGGGCTGAAAGGAGTTTCCCAAGAAGAAGTAGCAATGAT
GAATAAAATAAAAATGGAAGAAAATAAGCGTGAACTAGAAAAGGTGAAGAAACGTAGGATAGAACGTGAGTTAGAAA
AGCAGCAGCGCGAAGAGGAGATGTCCTTGATGCAACGGAGTAAAGAGGCTGCTCAGTTTCAGGAGTGGGAGCAACAC
GAAGACATGTTTCATCTTGAGCAAGCCCGTCTTCGATCGCGTATTCGTATCCAGGATGGCCGCGCAAAACCTATTGA
TCTTCTCGCCAAATACATCAGCACGGAAGAAGAAGTTGACGCTGTTGAAATGCACGAGCCGTATACATACCTTAATG
GTTTAACCATTAAAGATTTAGAAGATTTGGTTGCTGATATTAAGGTATACATAGAATTGGAAAGAGGGATGAACCTC
GACTATTGGAACGACATTACTGTAATTGTTGAAGATGAACTGTACAAACTGCGGAAACAAGACTCTCAAACGGAATA
TCAAGCAGCCGTTGCGAGGCGTGAGGGAATCCATGAATCAGTTGCCAAAGATGTTGCAAATGTACTGAAAGGCAAGA
CAGCCGAACAATTGGTGGCTCTTCAGACACAAATTGAAGCCAAACTGAAGAACAAAGCAGATGGAGTAGATATAGGA
TATTGGGAATCTCTACTTTCACAATTAAAAGCTCATCAAGCCAGAGCGCGTCTACGAGATCGACATCAAAACAATTT
GTTCAAAAAACTCCAAGTGCTCAAAGCAGAGCAAGGAGTTGACGAAAATTCCTCAGTGCCAAAGCAAGAAATCTCAG
ATGCTCCGTCCACCTCTGCTTCTTCGGCAAAACCAGAACGGCAAACTGAAACTCGGGGCAAAGAAGACACAGATGAT
TCATCTAATGAAGAGATGGATGAAGCAAAATTGGCGGAAGCAATGATAAACGAATGCATTGCAGAGTACGAGGCAGG
AGGTTACAGTCCAAAATACGTATCTCCATCCAGTCTTGAACCTGGAACAATCATCACCCTGGTAACAGAGGATTTGC
AACGGCTTGAATTCGCAAGGCGTAGAGTTCAAGGACTAGGAGCTAAAGTAGAGAATGTTATCACTGCAGAGGAAAAA
GCACTGCATCGTGAAGCAAGGAAAGGCATGACAGACGATGAGGTTGAATTCTCTGTTGAGTCCATACTCGAAAACCA
GGTTTACTTTTGGTCAGACAAATACCGTCCCAGGAAACCTAGATACTTCAACAGGGTGCACACTGGATTTGAGTGGA
ATAAATATAATCAAACTCATTACGATATGGATAATCCGCCTCCTAAAATTGTTCAAGGCTATAAATTCAACATTTTC
TACCCTGATCTGATAGATAAGTCATCAACTCCTGAATACTTTTTGACGCCTTGTCCTGACAATCATGATTTTGCCAT
CCTACGATTCCATGCTGGACCTCCATATGAAGACATCGCTTTCAAAATTGTAAATCGGGAATGGGAGTACTCATACA
AACGGGGATTCCGCTGTCAGTTCCAGAATAATATTTTCCAGCTATGGTTCCATTTCAAAAGATATCGTTACCGGAGG
TGA
