# Supplementary material for: Optimizing Efficient RNAi-Mediated Control of Hemipteran Pests (Psyllids, Leafhoppers, Whitefly): Modified Pyrimidines in dsRNA Triggers
Source: Plants (Basel). 2021 Aug 26;10(9):1782. doi: 10.3390/plants10091782 (PMC8472347; doi:10.3390/plants10091782)
Supplement: Supplementary file 1 [file plants-10-01782-s001.zip › plants-1322767-supplementary/plants-1322767-Supplemental Files Hunter/Supplemental TABLE_S2_HUNTER -Mean Number of Psyllid Eggs.pdf]

Optimizing Efficient RNAi-mediated Control of Hemipteran Pests (Psyllids and Whitefly): Modified pyrimidines in dsRNA Triggers.

Wayne Brian Hunter[1*] and William M. Wintermantel[2]

TABLE_S2_Mean Number of Psyllid Eggs, per Six Petiole 'Flush' per plant. Four potted citrus seedlings per treatment. Three experimental trials. One-way ANOVA. The ***F***-ratio value = 0.41535. The ***p***-value = 0.664761. The result was '*not significant*' at $P < 0.05$.

**EGGS**	**Mean Number of Eggs per plant; 4 plants/ treatment; with 6 new growth flush per plant**		
**Trial**	**Control**	**Soap**	**Tre-dsRNA**
T1-1	38.08	28.17	35.5
T1-2	39.08	51.21	36.83
T1-3	40.08	39.5	38.33
T2-1	41.08	27.93	37.33
T2-2	42.08	42.33	29.5
T2-3	43.08	39.67	53.33
T3-1	44.08	43.33	47.58
T3-2	45.08	43.33	44.98
T3-3	46.08	39.46	48.58
**Sum**	**378.75**	**354.93**	**371.96**
**Ave**	**42.08**	**39.44**	**41.33**
**Count**	**9**	**9**	**9**
**Var**	**6.67**	**48.52**	**52.02**
**SE**	**0.86**	**2.32**	**2.40**
