# Supplementary material for: Optimizing Efficient RNAi-Mediated Control of Hemipteran Pests (Psyllids, Leafhoppers, Whitefly): Modified Pyrimidines in dsRNA Triggers
Source: Plants (Basel). 2021 Aug 26;10(9):1782. doi: 10.3390/plants10091782 (PMC8472347; doi:10.3390/plants10091782)

Optimizing Efficient RNAi-mediated Control of Hemipteran Pests (Psyllids and Whitefly): Modified pyrimidines in dsRNA Triggers.

Wayne Brian Hunter[1]* and William M. Wintermantel[2]

Figure S1. Global Distribution Maps. CABI-Hemipteran Vectors

Asian citrus psyllid, *Diaphorina citri Kuwayama* https://www.cabi.org/isc/datasheet/18615

https://www.cabi.org/isc/datasheet/18615#totaxonomicTree

CABI-Distribution-MAP -- Asian citrus psyllid, *Diaphorina citri*,
https://www.cabi.org/isc/datasheet/18615#todistribution

Taxonomic Tree – *Diaphorina citri*
Domain: Eukaryota
Kingdom: Metazoa
Phylum: Arthropoda
Subphylum: Uniramia
Class: Insecta
Order: Hemiptera
Suborder: Sternorrhyncha
Unknown: Psylloidea
Family: Liviidae
Genus: *Diaphorina*
Species: *citri*

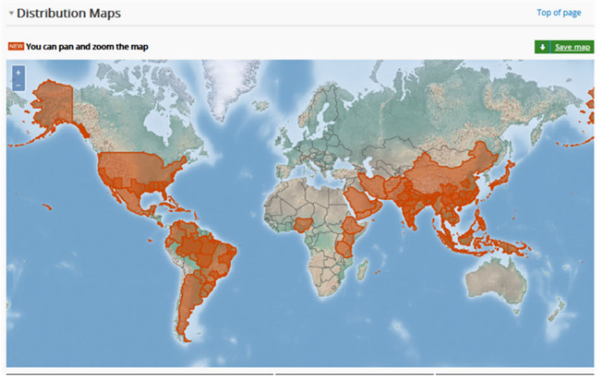

Whitefly, *Bemisia tabaci*

https://www.cabi.org/isc/datasheet/8927#totaxonomicTree

CABI –Distribution Map-Whitefly, *Bemisia tabaci*
https://www.cabi.org/isc/datasheet/8927#toDistributionMaps

Taxonomic Tree—Whitefly, *Bemisia tabaci*
Domain: Eukaryota
Kingdom: Metazoa
Phylum: Arthropoda
Subphylum: Uniramia
Class: Insecta
Order: Hemiptera
Suborder: Sternorrhyncha
Unknown: Aleyrodoidea
Family: Aleyrodidae
Genus: *Bemisia*
Species: *tabaci*

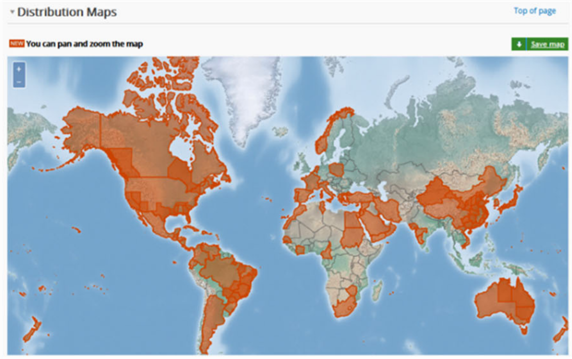

Glassy-winged Sharpshooter, *Homalodisca vitripennis* https://www.cabi.org/isc/datasheet/27561

Taxonomic Tree –*Homalodisca vitripennis*
Domain: Eukaryota
Kingdom: Metazoa
Phylum: Arthropoda
Subphylum: Uniramia
Class: Insecta
Order: Hemiptera
Suborder: Auchenorrhyncha
Unknown: Cicadelloidea
Family: Cicadellidae
Genus: *Homalodisca*
Species: *vitripennis*

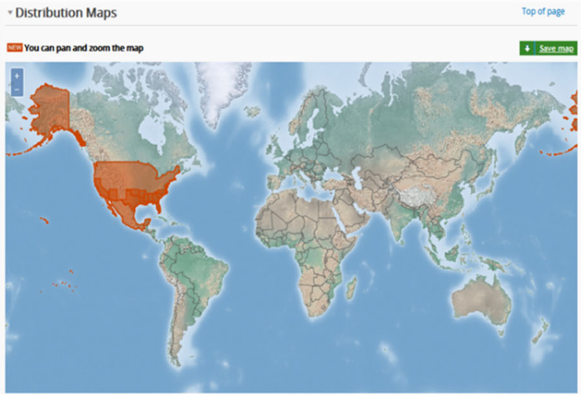

Supplement: Supplementary file 1 [file plants-10-01782-s001.zip › plants-1322767-supplementary/plants-1322767-Supplemental Files Hunter/Supplemental_FIGURE S1. HUNTER Global Distribution Maps- Vectors.pdf]
