# Supplementary material for: Optimizing Efficient RNAi-Mediated Control of Hemipteran Pests (Psyllids, Leafhoppers, Whitefly): Modified Pyrimidines in dsRNA Triggers
Source: Plants (Basel). 2021 Aug 26;10(9):1782. doi: 10.3390/plants10091782 (PMC8472347; doi:10.3390/plants10091782)
Supplement: Supplementary file 1 [file plants-10-01782-s001.zip › plants-1322767-supplementary/plants-1322767-Supplemental Files Hunter/Supplemental_FIGURE S2_HUNTER- Soluble Trehalase-1 dsRNA sequences for Diaphorina citri.pdf]

Optimizing Efficient RNAi-mediated Control of Hemipteran Pests (Psyllids and Whitefly): Modified pyrimidines in dsRNA Triggers.

Wayne Brian Hunter[1]* and William M. Wintermantel[2]

FIGURE_S2. ***Soluble Trehalase-dsRNA*** **regions tested for activity after ingestion by adult Asían Citrus Psyllid, (Hemiptera: Liviidae).**

FIGURE S2. Soluble *Trehalase-1*-dsRNA-1. (SEQ ID NO: 1, in Hunter, Gonzalez, Andrade et al, 2019).

AGTATACGGGCGACACCAACTTTATAAGAACCCACCTTAAGTCACTAACCAACGAGTTTGAATACTGGAT
GAAGAGACATATGGTCACTGTAGAGAAAAATGGCAAGTACTACACCATGGCTCGATACTACGCTCCGTCC
AGAGGCCCTAGGCCCGAGTCTTACAGAGAGGACTACCATGAGGCAGCAGATTTGCAGACAGAGGATGAGA
AAAACTTCCTGTACTCAGAGCTGAAGGCAGGTGCCGAAACCGGATGGGACTTTTCCAGTCGGTGGTTCAT
CGCACGGGACGGTAGCAATAGAGGAGGCCTCAAATACATTCGCACCACATCGATCATTCCCGTGGACCTC
AATGCGATCCTTCAGATGAACGCTAACTATCTGAGCGAATGGTGGCTCAAATTTGGCAACAAGGATTTGA
GTGCCAAGTACAAGAAGATTGCGTACCAA**CTGCTTGAAGCCATTCATGA**

FIGURE S2. Soluble *Trehalase*-dsRNA- 2. (SEQ ID NO: 6, in Hunter, Gonzalez, Andrade et al, 2019)

TGAGCTGAGGACGATTGATG**ATTTCAGCCAAATCTACT**GCAAGGGAGAACTTCTGGACAAGGTACAGCGA
GGAAACGTGTTTCCAAACGACTCGAAATCGTTTGTCGATCTCAAACTGAAACAGCCAGAGGACGTGATTC
TGGCCAAGTTCCGAGCCTTGCTCACCAATAATGCTGATCCCGACACCACCACACTGACCAACTTTGTCAA
CGAATACTTTGAAGCAG*GCAATGAGCTGCAAGTCTGGAGTCCTCCAGATTTCACCTCCAACCCGAGTATC*
GAGAACAAAATCTCCGACGCCAAATACAGACAGTTTGCCCTCGACCTGAACCAAATTTGGAAAGAGTTGG
*GCCGCATAGTAAAACAAGATGTAAGGGACAACCCTCAACTGTACTCACTCATATACACACCCAATGGA*A***TT***
CTTCATTCCTGGAGGACG

FIGURE S2. Soluble *Trehalase-1*-dsRNA-3. (SEQ ID NO: 11, in Hunter, Gonzalez, Andrade et al, 2019))

TCAATGCGATCCTTCAGATGAACGCTAACTATCTGAGCGAATGGTGGCTCAAATTTGGCAACAAGGATTT
GAGTGCCAAGTACAAGAAGATTGCGTACCAACTGCTTGAAGCCATTCATGAGGTTCTATGGAATGAACAG
GTTGGTGTATGGCTAGACTACGACATTAAGAACAAGAAGCCCCGAAATTATTTCTACGTCTCAAACATAA
CTCCTCTGTGGACATTGAGCTACAAATTCTCCAAACAATATGTGGCTGAGAGAGTACTGCAGTATTTGCG
AGACAATGAAATCATCACCAAGGACAATCAAGTGAAATTCTATGGTACCCCTACCTCCTTGTTCAACTCT
ACTCAACAATGGGATTACCCTAATGCCTGGGCCCCACTACAGGCATTCATCATACAAGGCTTGGACTACA
CGCAAGACAAATTAGCAAAGCAAGTGGCATACCGACTGGCTGAAAAGTGGCTCTTCACAAACTATATGGG
CTATGAAACTAGCAAGGCTATGTTTGAGAAATATGATGTAGAACTCATTGGAAAGACAGGTAATGGAGGT
GAGTACGAGGCACAAACTGGATTTGGTTGGACCAATGGATTCGCATTTGAGCTTCTAAATAGATACGGAA
AAACTATATCTTTCAACAATACTCAAGGAAGCTACTACAATAAAATCCCCGGATCCGGTTACTTATCCGG
CTATTATCCG**TCTTTCATGTCCGGAAGACC**
