# Supplementary material for: Optimizing Efficient RNAi-Mediated Control of Hemipteran Pests (Psyllids, Leafhoppers, Whitefly): Modified Pyrimidines in dsRNA Triggers
Source: Plants (Basel). 2021 Aug 26;10(9):1782. doi: 10.3390/plants10091782 (PMC8472347; doi:10.3390/plants10091782)

Optimizing Efficient RNAi-mediated Control of Hemipteran Pests (Psyllids and Whitefly): Modified pyrimidines in dsRNA Triggers.

Wayne Brian Hunter[1]* and William M. Wintermantel[2]

FIGURE S3. Phylogeny of the *Syntaxin 1A* mRNA sequence, from *Diaphorina citri*, Asian citrus Psyllid. Analyses using BLASTx to Hemiptera, (NCBI, nr database, setting 'Somewhat Similar', accessed June 07 2021). Selected top returns, Includes the predicted *Syntaxin 1A* in *Bemisia tabaci.* Top related Taxa to Psyllid *Syntaxin 1A*, shown includes: Aphids, Planthopper, and Whitefly.

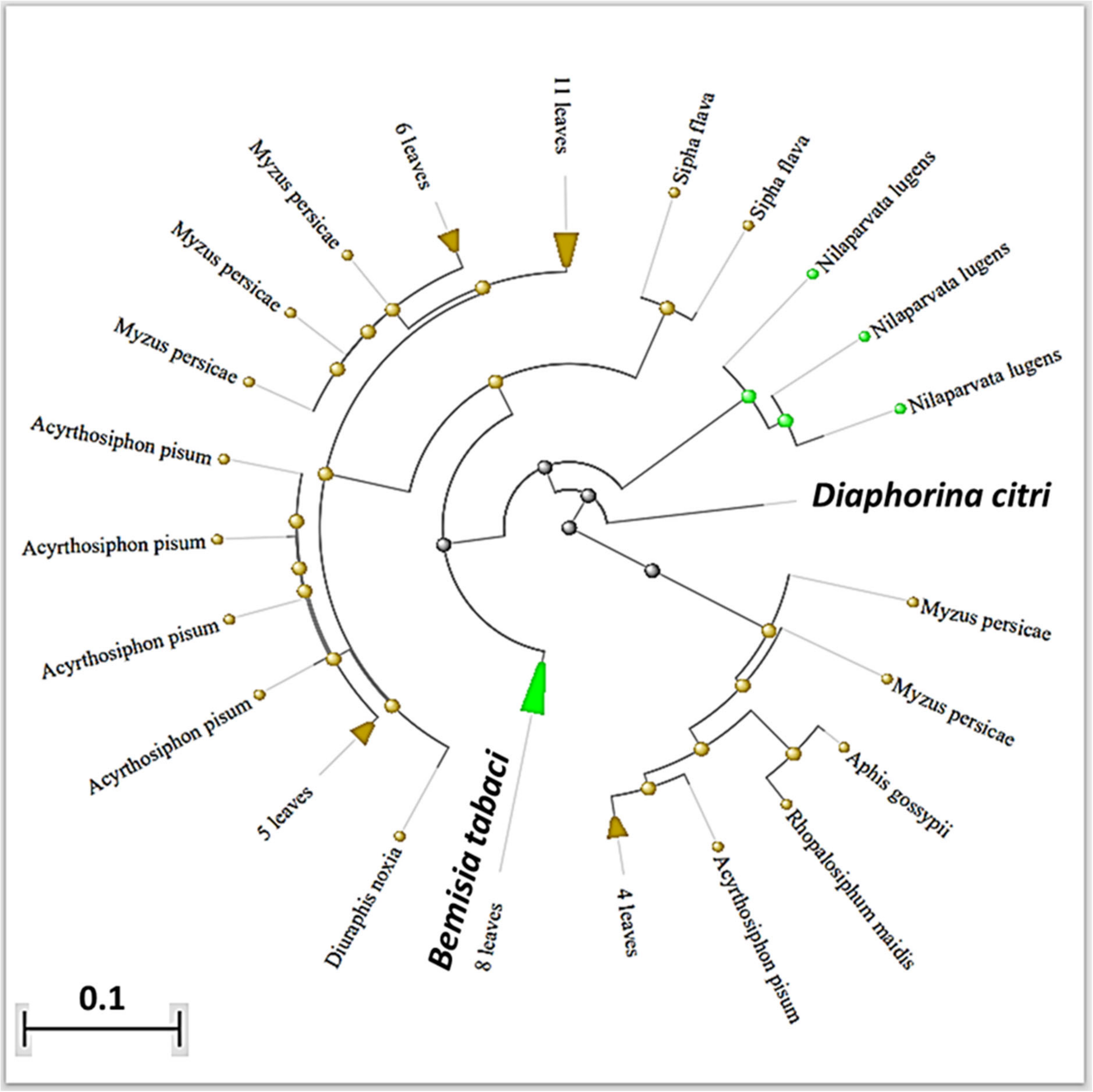

Supplement: Supplementary file 1 [file plants-10-01782-s001.zip › plants-1322767-supplementary/plants-1322767-Supplemental Files Hunter/Supplemental_FIGURE S3_HUNTER-Phylogeny Syntaxin Diaphorina citri to Hemiptera.pdf]
