# Supplementary material for: Optimizing Efficient RNAi-Mediated Control of Hemipteran Pests (Psyllids, Leafhoppers, Whitefly): Modified Pyrimidines in dsRNA Triggers
Source: Plants (Basel). 2021 Aug 26;10(9):1782. doi: 10.3390/plants10091782 (PMC8472347; doi:10.3390/plants10091782)
Supplement: Supplementary file 1 [file plants-10-01782-s001.zip › plants-1322767-supplementary/plants-1322767-Supplemental Files Hunter/Supplemental_FIGURE-S5 Modification Oligos and Propterties_HUNTER.pdf]

**Optimizing Efficient RNAi-mediated Control of Hemipteran Pests (Psyllids and Whitefly): Modified pyrimidines in dsRNA Triggers.**

Wayne Brian Hunter[1]* and William M. Wintermantel[2]

FIGURE S5. Modification in oligonucleotides and their properties (modelled *after:* Glazier et al, 2020).

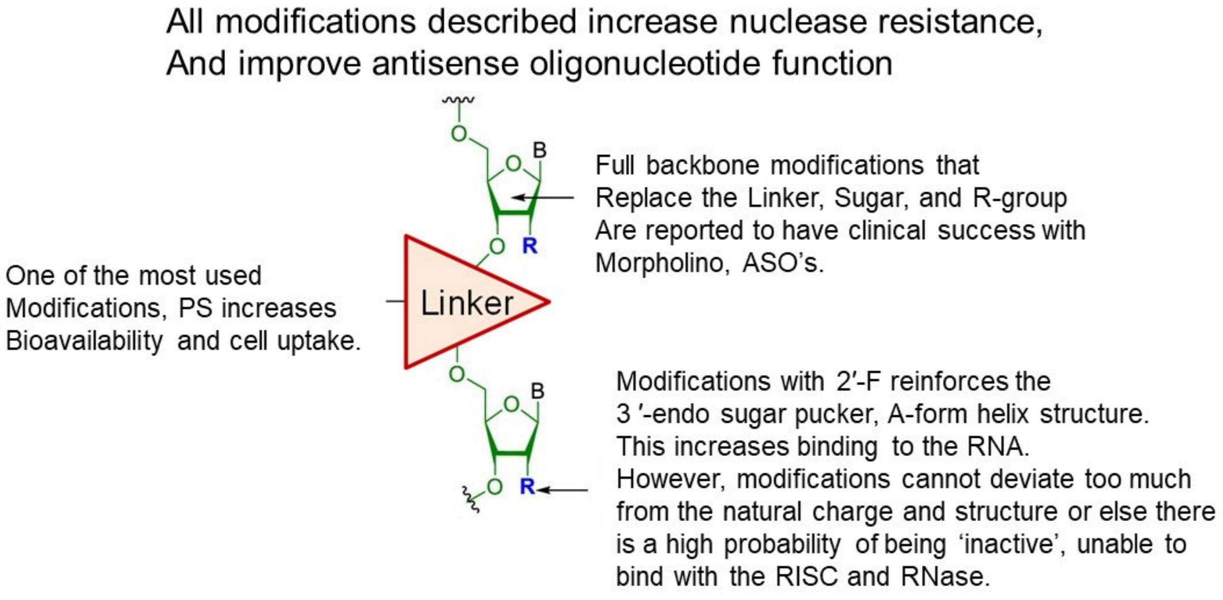


**After Review by: Glazier,** D.A., Liao, J., Roberts, B.L., Li, X., Yang, K., et al, (2020). Chemical synthesis and biological application of modified oligonucleotides. Bioconjug. Chem. 31: 1213–1233. Doi:10.1021/acs.bioconjchem.0c00060.
