# Supplementary material for: Optimizing Efficient RNAi-Mediated Control of Hemipteran Pests (Psyllids, Leafhoppers, Whitefly): Modified Pyrimidines in dsRNA Triggers
Source: Plants (Basel). 2021 Aug 26;10(9):1782. doi: 10.3390/plants10091782 (PMC8472347; doi:10.3390/plants10091782)
Supplement: Supplementary file 1 [file plants-10-01782-s001.zip › plants-1322767-supplementary/plants-1322767-Supplemental Files Hunter/Supplemental_S7_HUNTER Gene Data-mining from De Novo Genomes.pdf]

Optimizing Efficient RNAi-mediated Control of Hemipteran Pests (Psyllids and Whitefly): Modified Pyrimidines in dsRNA Triggers.

Wayne Brian Hunter* and William M. Wintermantel

S7. Report: Gene Data-mining from *De Novo* Genomes (Psyllid, Whitefly, Leafhopper)

***Gene target selection Rationale*:**

Insect genomes and transcriptomes provide the foundational information needed to design effective RNAi pest management. The development and use of the psyllid, whitefly, and leafhopper genomes were multi-institutional efforts that produced open-source data sets and are described. The gene targets in this study were selected after in-depth literature searches from studies reporting genes with a critical function in embryo and larvae development from studies in mammals (Bult et al, 2019; Cerezo et al, 1995; Yanay et al, 2008), nematode, *Caenorhabditis elegans* (*see e.g. and references therein:* Zipperlen et al, 2001; Sonnichsen et al, 2005) and insects, like *Drosophila*; and *Tribolium* (*see e.g. and references therein*: Gu and Knipple 2013; Gelbart et al, 1997; Schmitt-Engel et al, 2015; Ulrich et al, 2015, Yanay et al, 2008). The sequences were assembled from *de novo* genomes, transcriptomes, or from the Sequence Read Archive (SRA) deposited by the Genome Consortiums of each insect (NCBI database) https://www.ncbi.nlm.nih.gov/sra/docs/) followed with validation by resequencing from each insect vector.

Research has shown that trehalase functions in hemipteran development and survival (Chen et al, 2010; Gu et a;. 2009; Tang et al, 2017; Yu et al, 2020; Xu et al, 2009). When Trehalase is suppressed then the synthesis of trehalose is reduced and there is an increase in psyllid mortality (Liu et al, 2020), with similar reports in planthoppers (Yang et al, 2017) and other invertebrates (Tang et al, 2018; Shukla et al, 2018). *Trehalose* is the main blood sugar of insects, and the enzyme trehalase is involved in energy metabolism and regulates trehalose levels in cells. The two forms of trehalase in psyllid (soluble and membrane bound) and their corresponding genes were identified from data mining the *D. citri*, psyllid pathway network

dataset, of the *Diaci_v2,genome* and the official gene set (OGS-v2, 2019) (open access at: www.citrusgreening.org). BLAST analyses with BLASTx and BLASTp (NCBI, and ExPASY tools, https://web.expasy.org/translate/) identified sequences. The soluble *Trehalase-1b,* mRNA sequence, *Trehalase* (EC:3.2.1.28) and regions for dsRNA development are shown in the Supplemental Materials. Phylogenetic trees for the putative proteins for the psyllid *Trehalase*, and *Syntaxin 1A* in psyllid, *D. citri* and whitefly, *B. tabaci*, and the *Cactin* sequence (*D. citri, B. tabaci, and H. vitripennis*) were compared using BLASTn , BLASTx, and BLASTp to the Order: Hemiptera, online at NCBI (June 10, 2021). (Figures of phylogenetic trees for *Trehalase*-1 in *Diaphorina citri*, and *Syntaxin 1A* in *Bemisia tabaci*, to hemipterans, Supplemental Materials).

Cactin, is a conserved protein that interacts with the IkappaB protein Cactus and modulates its function (Lin et al, 2000). The IκB inhibitor Cactus functions in promoting dorsal nuclear localization and activity in the insect embryo for proper development (Cardoso et al, 2017).

Syntaxin 1A, interacts with multiple exocytic proteins to regulate neurotransmitter release, (Wu et al, 1999), and modulates sexual maturity rates and progeny egg size related to phase changes in locusts (Chen et al, 2015), while providing guidance in a conserved role for pre- and post-commissural midline axonal formation in flies and other insects (Ros et al, 2018).

***Genomics, Bioinformatics, Datasets*:**

Genes selected were data mined and assembled from raw datasets, and then re-sequenced and manually annotated (Hunter et al, 2009; Reese et al, 2013; Chen et al, 2016). Public release of an improved ACP genome, DIACI_2.0, and Official Gene Set, DIACI_OGS_v2 (Saha et al., 2017a-f), was possible in part with support from National Institute of Food and Agriculture, USDA, Specialty Crops Research Initiative/Citrus Disease Research & Extension, Award #2015-70016-23028, "*Developing an Infrastructure and Product Test Pipeline to Deliver Novel Therapies for Citrus Greening Disease*', Lead PI: Prof. Dr. Susan Brown, (Kansas State University, Manhattan, KS); CoPI's: Bioinformatics and Annotation, Drs: Surya Saha, and Lukas Mueller, (Boyce Thompson Institute, NY); CoPI: Prof. Dr. Tom D'Elia, Gene Annotation Teams in Florida, , Indian River

State College, Ft. Pierce, FL; CoPI: Dr. Michelle Heck , Proteomics, USDA,ARS, Cornell, NY; CoPI: Prof. Dr. Carolyn Slupsky, Department of Food Science & Technology, Univ. California-Davis, CA; CoPI: Dr. Robert Shatters, Molecular biology, USDA, ARS, Fort Pierce, FL. All Psyllid Data are available at: https://citrusgreening.org/organism/Diaphorina_citri/genome and at: www.citrusgreening.org. Genes identified by *in silico* comparisons, BLASTn, BLASTx, and with the published literature on genes critical for insect development, glycolysis, gluconeogenesis, and trehaloneogenesis, chitin production, and survival were validated by resequencing (First International Psyllid Genome Consortium, IPGC 2009 (Hunter, USDA,ARS/ Saha, S., Mueller, L., Boyce Thompson Institute, NY): Diaci database, Official Gene Set_v3; Diaci_v2.0 genome [www.citrusgreening.org]; *Diaphorina citri* MCOT transcriptome https://data.nal.usda.gov/dataset/diaphorina-citri-mcot-transcriptome; Data mining started with *Diaphorina citri* genome assembly Diaci 1.9 (2011-2014) https://data.nal.usda.gov/dataset/diaphorina-citri-genome-assembly-diaci-19, Diaci Bioproject: PRJNA609978 initiated at NCBI, https://www.ncbi.nlm.nih.gov/bioproject/609978) (Hunter et al, 2009). Psyllid Annotation Team in Florida led by Prof. Dr. Tom D'Elia, (IRSC, Ft. Pierce, FL), with student annotators at University of Cornell, NY, University of California-Davis, and Kansas State University, which conducted manual annotations of selected genes in critical pathways to provide high quality gene models required for designing molecular based therapeutics like RNA interference (RNAi) (Saha et al, 2017a,b,c;d,e,f; 2019; Chen et al., 2016; Miller et al, 2020a,b,c; 2021). Annotated orthologs of genes involved in the important metabolic pathways of: Glycolysis, Gluconeogenesis, and Trehaloneogenesis, were compared to each insect pest *D. citri* (Florida colony, USA Hall et al, 2013), *B. tabaci* (MEAMI, Tanzania, Chen et al, 2016; 2019), *H. vitripennis* (Hunter et al, 2006).

The International leafhopper genome consortium was initiated in 2009 (Hunter, USDA, ARS), producing expressed transcripts from adults, midgut, ovaries, and testes. The project was incorporated into the i5k Glassy-winged Sharpshooter Leafhopper genome, *Homalodisca vitripennis* (Initiated Hunter 2009, USDA, ARS. prev. *H. coagulata*) (Ag Data Commons, https://data.nal.usda.gov/dataset/homalodisca-vitripennis-genome-annotations-v053). The International Whitefly Genome consortium, conference in 2010 [McKenzie, USDA, ARS]

(Leshkowitz et al, 2006] set out plans to produce genomes from whitefly biotypes (Wang et al, 2010). The first whitefly genome completed in 2015 (Chen et al, 2016). The genome assembly for the whitefly Database (http://www.whiteflygenomics.org/cgi-bin/bta/index.cgi), was led by Dr. Zhangjun Fei, (Genomics and Systems, at Boyce Thompson Institute, Cornell, NY). The International initiative was a collaboration with: the Office of International Research projects, OIRP, the USDA, ARS (Drs. Bill Wintermantel, Kai Ling, Wayne Hunter), and The Danforth Center, the USAID-funded programs and The Bill & Melinda Gates Foundation's Great Lakes Cassava Initiative (GLCI), Dr. James Legg, (International Institute of Tropical Agriculture, IITA, Plant Health Scientist, Dar es Salaam, Tanzania), and Dr. Leena Tripathi, (Plant Biotechnology, Director of East Africa Hub, IITA-Kenya representative, Dar es Salaam, Tanzania).

Selecting target genes in psyllids: The International Asian Citrus Psyllid Genome Consortium established in 2009 (Hunter et al, 2009; Reese et al, 2013; Hunter and Reese 2014). From the international effort the genome, transcriptome, and Official Gene Set, of the Asian Citrus Psyllid vector, *D. citri*, were completed with manual annotations of important gene families (2014 thru 2021) (Saha et al, 2017a,b,c;d,e,f; 2019; Macias-Velasco et al, 2018; Hosmani et al. 2019;) (Genome Diaci_v2.0, and OGS_v3.0, available at: www.citrusgreening.org). The *D. citri* genome is the reference genome for psyllids, guiding analyses of other psyllid vectors of 'Candidatus Liberibacter solanacearum', CLso, specifically the tomato psyllid *Bactericera cockerelli* (Tang et al, 2020). Most insects lack the final enzyme in the gluconeogenesis pathway. Thus, in insect trehaloneogenesis, the glucose-6-phosphate is converted into trehalose-by-trehalose 6-phosphate synthase (TPS). The Trehalase enzymes then degrade trehalose into two glucose molecules (Shukla et al, 2015). In insects there are three enzymes involved in Trehaloneogenesis: the trehalose-6-phosphate synthase (TPS), trehalose-6-phosphate phosphatase (TPP), and trehalase (TREH) (Matsuda et al, 2015; Liu et al, 2020; Miller et al, 2020a,b,c; 2021). TPS catalyzes the transfer of glucose from UDP-glucose to G6P forming trehalose 6-phosphate (T6P) and UDP (Tang et al, 2017; 2018). Two copies of TPS were annotated in *D. citri* and were found to be highly expressed in the abdomen compared to other tissues (Yu et al, 2020; Saha, et al, 2017).

In trehaloneogenesis, the two TREH models showed low to moderate expression in both males and females, and TPS was found to be highly expressed in the abdomen compared to other body tissues in *D. citri*. (Liu et al, 2020; Yu et al, 2020), similar to planthoppers (Chen et al, 2010; Gu et al, 2009; Yang et al, 2017). Orthologous protein sequences for the glycolysis, gluconeogenesis, and trehaloneogenesis pathways were obtained from the NCBI protein database. These FASTA sequences were BLAST searched in the *D. citri* MCOT protein database (www.citrusgreening.org) to find predicted protein models (Saha et al, 2019; Hosmani et al, 2019). The MCOT IDs of the predicted protein models were loaded into Apollo, consisting of the 2.0 and 3.0 versions of the *D. citri* genome, to search for and curate gene models. Gene models were curated using evidence tracks, such as de novo transcriptome, MCOT gene predictions, RNA-Seq, Iso-seq, and ortholog, to support and evaluate gene structure (Saha et al, 2017a,-f; 2019; Hosmani et al, 2019; Macias et al, 2018; Massimino et al, 2020; Miller et al, 2020a,b,c; 2021; Vosburg et al, 2020; Yang et al, 2020). The protein sequences from the curated gene models were analyzed with BLASTp at NCBI to identify appropriate domains, and compare the sequence identity and query coverage to other insects within the Hemiptera. A neighbor-joining phylogenetic tree of the annotated carbohydrate metabolism genes in *D. citri* and orthologous sequences were created through MEGA using MUSCLE multiple sequence alignment with p-distance for determining branch length and 1,000 bootstrap replicates (Miller et al, 2020a,b;c; Saha et al, 2019; Hosmani et al, 2019). The psyllid genes and datasets are openly available at: (ftp://ftp.citrusgreening.org/annotation/MCOT/) (Saha et al, 2017a). Transcripts from Maker, Cufflinks, Trinity and Oases were translated to proteins with Transdecoder version 2.0.1 and unique proteins were kept (Reese et al, 2013; Saha, et al, 2017a-e; 2019)..

Using the genomic data, a method was developed using CRISPR/Cas9 to produce the first heritable gene edits in the Asian citrus psyllid (Hunter et al, 2018). The new method used adult female psyllids with microinjection of the CRISPR components near the ovaries. The method even worked when nymphs. The method was improved using Branched Amphiphilic capsules that self-assemble in aggregations around negatively charged RNA and DNA sequences. The improved method increased the success of producing mutants in the G2 from 12% t over 40%. Injected Go females, produced mutant G1's that were paired with wild-type which resulted in

production of G2 mutants, as a rate of about 2-6%. The low survival was due to the critical gene selected for the knock-out. The guides were made to the predicted: *Diaphorina citri* thioredoxin-2-like (LOC103521994) gene, for transcript variant X1 (sequence ID: XM_008487100.1) the fragment length of 1243 bp as described in genomic assembly DIACI_v2.0: https://citrusgreening.org/organism/Diaphorina_citri/genome. The production of the multiple sgRNAs was performed by Dharmacon Inc. (Lafayette, CO) (http://dharmacon.horizondiscovery.com/gene-editing/crispr-cas9/#all). The psyllid TRX-2 was selected for knockout, and produced a deletion of 556nt. The biological effects were adults with short lifespans (6 - 8d), and significantly reduced fecundity approx.. 3 eggs per adult female lifespan (Hunter et al, 2019a,b).

References:

Chen, W., Hasegawa, D.K., Kaur, N., Kliot, A., Pinheiro, P.V., Luan, J., et al. The draft genome of whitefly *Bemisia tabaci* MEAM1, a global crop pest, provides novel insights into virus transmission, host adaptation, and insecticide resistance. *BMC Biol.* 2016, 14, 110. https://doi.org/10.1186/s12915-016-0321-y

Chen, Q., He, J., Ma, C., Yu, D., Kang, L. *Syntaxin 1A* modulates the sexual maturity rate and progeny egg size related to phase changes in locusts. *Insect Biochem. Mol. Biol.* 2015, 56, 1-8. Doi:10.1016/j.ibmb.2014.11.001.

Chen, W., Wosula, E.N., Hasegawa, D.K., Casinga, C., Shirima, R.R., Fiaboe, K.K.M., et al. Genome of the African cassava whitefly *Bemisia tabaci* and distribution and genetic diversity of cassava-colonizing whiteflies in Africa. *Insect Biochem. Mol. Biol.* 2019, 110, 112–120. Doi: 10.1016/j.ibmb.2019.05.003

Chen, J., Zhang, D., Yao, Q., Zhang, J., Dong, X., Tian, H., et al., (2010). Feeding-based RNA interference of a trehalose phosphate synthase gene in the brown planthopper, *Nilaparvata lugens*. *Insect Mol. Biol.* 2010, 19, 777–786. Doi:10.1111/j.1365-2583.2010.01038.x

Citrus Greening Solutions (2020). Annotation of psyllid genome (2018-2020). https://citrusgreening.org/annotation/index . Accessed 08 March 2021.

Hasegawa, D.K., Chen, W., Zheng, Y., Kaur, N., Wintermantel, W.M., Simmons, A.M., Fei, Z., Ling, K.S. Comparative transcriptome analysis reveals networks of genes activated in the whitefly, *Bemisia tabaci* when fed on tomato plants infected with Tomato yellow leaf curl virus. *Virology* 2018, 513, 52-64. Doi:10.1016/j.virol.2017.10.008.

Hunter, W.B. (2006). Insect/Arthropod Genomics Workshop, founder/ organizer. International Plant & Animal Genomes XIV Conference, San Diego, CA. (2005-2018). (*Current organizer*: Dr. Marce Lorenzen 2019-2022).

Hunter, W.B., Dowd, S.E., Katsar, C.S., Shatters, R.G., McKenzie, C.L., Hall, D.G. (2009). Psyllid biology: Expressed genes in adult Asian citrus psyllids, *Diaphorina citri* Kuwayama. *The Open Entomol. Jour*. 2009, 3, 18–29. https://www.ars.usda.gov/ARSUserFiles/11768/2009Hunter_TOENTOJ.pdf

Hunter, W.B., Gonzalez, M.T., Tomich, J. BAPC-assisted CRISPR/Cas9 system: Targeted delivery into adult ovaries for heritable germline gene editing (Arthropoda: Hemiptera). bioRxiv_2018 [Preprint]. http://dx.doi.org/10.1101/478743

Hunter, W.B., Gonzalez, M.T., Tomich, J. BAPC-assisted-CRISPR-Cas9 Delivery into Nymphs and Adults for Heritable Gene Editing (Hemiptera). *ASBMB,* Orlando, FL, 2019. https://www.fasebj.org/doi/abs/10.1096/fasebj.2019.33.1_supplement.626.2

Hunter, W.B., Gonzalez, MT., Tomich, J. BAPC-assisted-CRISPR-Cas9 Delivery into Adult Psyllid Ovaries and Pupae for Heritable Gene Editing (Hemiptera). Peer-Reviewed: *Research Highlights,* 2019. https://citrusgreening.org/disease/researchhighlights/index

Hunter, W.B. and Katsar, C.S. Gene expression in two leafhopper vectors of Pierces Disease of grapes, Glassy-winged sharpshooter and Blue-green sharpshooter (Hemiptera: Cicadellidae). 2006, DS-11. *Joint Proceed. 89th Annual meeting and 6th International Caribbean Conf. Florida Entomol. Soc*. http://www.flaentsoc.org/WH%20FES%2006%202LH.pdf

Hunter, W.B., Katsar, C.S., McKenzie, C.L., Shatters, R.G., Weathersbee, A.A., D.G. Hall. Gene expression in the Asian citrus psyllid: Vector of citrus greening (Hemiptera: Psyllidae). 2006, DS-10. *Joint Proceedings of the 89th Annual meeting and 6th International Caribbean Conference of Florida Entomol. Soc*. http://www.flaentsoc.org/fes06-dsp10.pdf

Hunter, W.B., Johnson, S., Rhodes, A., Davis, P., Christenson, M.K., Dang, P., Hunnicutt, L., Katsar, C.S., Dowd, S.E., Reese, J., McCarthy, J., Puterka, G., Reinke, M., Costa, H., et al. History of *Homalodisca coagulata*, (GWSS) Genomic data, ESTs, Transcriptomes, Assemblies. Early years.

2000-2012. *Ag Data Commons*, 2012. https://data.nal.usda.gov/dataset/history-hvit-genomic-data-ests-transcriptomes-assemlbies-annotations-early-years-2000-2010_5145

Kaur, N., et al., Transcriptome analysis of the whitefly, *Bemisia tabaci* MEAM1 during feeding on tomato infected with the Crinivirus, Tomato chlorosis virus, identifies a temporal shift in gene expression and differential regulation of novel orphan genes. *BMC Genomics* 2017, 18, 370.

Leshkowitz, D., Gazit, S., Reuveni, E., Ghanim, M., Czosnek, H., McKenzie, C., et al. Whitefly (*Bemisia tabaci*) genome project: analysis of sequenced clones from egg, instar, and adult (viruliferous and non-viruliferous) cDNA libraries. *BMC Genomics* 2006, 7, 79. Doi:10.1186/1471-2164-7-79.

Marchler-Bauer, A., et al. CDD/SPARCLE: functional classification of proteins via subfamily domain architectures. *Nucleic Acids Res*. 2017, 45(D), 200-3.

Marchler-Bauer, A., et al. CDD: NCBI's conserved domain database. *Nucleic Acids Res.* 2015, 43(D), 222-6.

Marchler-Bauer, A., et al. CDD: a Conserved Domain Database for the functional annotation of proteins. *Nucleic Acids Res*. 2011, 39(D), 225-9.

Marchler-Bauer, A., and Bryant, S.H. CD-Search: protein domain annotations on the fly. *Nucleic Acids Res*. 2004, 32(W), 327-331.

Macias-Velasco, J.F., Brunson, R.T., Hunter, W.B., Bextine, B.R. Evaluation of North American Asian citrus psyllid (Hemiptera: Liviidae) population genetics using cytochrome P450 melt curve analysis. bioRxiv, [Pre-Print],2018, 445734. Accessed April 23, 2021. https://doi.org/10.1101/445734

Massimino, C., Vosburg, C., Shippy, T., Hosmani, P.S., Flores-Gonzalez, M., et al. Annotation and analysis of yellow genes in *Diaphorina citri,* vector for the Huanglongbing disease. bioRxiv, [Pre-Print], 2020.12.22.422960. Accessed April 23, 2021. https://doi.org/10.1101/2020.12.22.422960

Miller, S., Shippy, T.D., Hosmani, P.S., Flores-Gonzalez, M., Mueller, L.A., et al. Segmentation pathway genes in the Asian citrus psyllid, *Diaphorina citri*. bioRxiv, [Pre-Print], 2020.12.24.424320. Accessed April 23, 2021. https://doi.org/10.1101/2020.12.24.424320

Miller, S., Shippy, T.D., Tamayo, B., Hosmani, P.S., Flores-Gonzalez, M., Mueller, L.A., et al. Chitin biosynthesis genes in *Diaphorina citri*, Asian citrus psyllid. bioRxiv, [Pre-Print], 2020.09.22.309211. Accessed April 23, 2021. https://doi.org/10.1101/2020.09.22.309211

Miller, S., Shippy, T.D., Tamayo, B., Hosmani, P. S., Flores-Gonzalez, M., Mueller, L.A., et al. Characterization of chitin deacetylase genes in the *Diaphorina citri* genome. bioRxiv, [Pre-Print], 2020.12.22.424074. Accessed April 23, 2021. https://doi.org/10.1101/2020.12.22.424074

Miller, A., Shippy, T.D., Tamayo, B., Hosmani, P. S., Flores-Gonzalez, M., Mueller, L.A., et al. Annotation of chitin biosynthesis genes in *Diaphorina citri*, the Asian citrus psyllid, *Gigabyte* 1, 2021. https://doi.org/10.46471/gigabyte.23

Miller, S., Shippy, T.D., Tamayo, B., Hosmani, P.S., Flores-Gonzalez, M., Mueller, L.A., Hunter, W.B., Brown, S.J., D'Elia, T., Saha, S. Supporting data for "Annotation of chitin biosynthesis genes in *Diaphorina citri*, Asian citrus psyllid". *GigaScience* Database 2021; http://dx.doi.org/10.5524/100900

Reese, J, Christenson, M.K, Leng, N., Saha, S., Cantarel, B., Lindeberg, M., et al. The Asian citrus psyllid transcriptome. *J. Genomics* 2013, (2), 54-58. Doi:10.7150/jgen.7692.

Saha, S., Cao, X., Flores, M., Jiang, H., Mueller, L.A. *Diaphorina citri* MCOT transcriptome. *Ag Data Commons*, 2017. https://doi.org/10.15482/USDA.ADC/1342726. Accessed 2021-07-09. (dataset).

Saha, S., Hosmani, P.S., Flores-Gonzalez, M., Hunter, W., D'Elia, T. Biocuration and improvement of the *Diaphorina citri* draft genome assembly with long reads, optical maps and long-range scaffolding. International Psyllid Annotation Consortium, Brown, S. and L.A. Mueller. 2017, 13, 27-28. *Proc. 10th Arthropod Genomics Symposium*. June 8–11, Notre Dame, IN. USA. http://globalhealth.nd.edu/assets/238736/ags_booklet.pdf

Saha, S., Hosmani, PS., Villalobos-Ayala, K., Miller, S., Shippy, T., Flores, M., et al. (43). Improved annotation of the insect vector of citrus greening disease: Biocuration by a diverse genomics community. 2017, *Database* 2017:bax032. https://doi.org/10.1093/database/bax032

Saha, S.; Hosmani, P; Villalobos-Ayala, K; Miller, S, Shippy, T., Flores, M., et al. *Diaphorina citri* Official Gene Set v1.0. *Ag Data Commons. 2017.* http://dx.doi.org/10.15482/USDA.ADC/1345524

Saha, S., Hunter, W., Mueller, L., Brown, S. *Diaphorina citri* genome assembly DIACI 1.9. *Ag Data Commons*, 2017. http://dx.doi.org/10.15482/USDA.ADC/1342727

Saha, S., Hosmani, P.S., Villalobos-Ayala, K., Miller, S., Shippy, T., Flores, M., et al. Improved annotation of the insect vector of citrus greening disease: Biocuration by a diverse genomics community. *Database*: *The Journal of Biological Databases and Curation*. 2019. https://doi.org/10.1093/database/baz035

Saha, S., Hunter, W.B., Reese, J., Morgan, J.K., Marutani-Hert, M., Huang, H., et al. Survey of endosymbionts in the *Diaphorina citri* metagenome and assembly of a *Wolbachia,* wDi draft genome. *PLoS One* 2012, 7(11), e50067. Doi:10.1371/journal.pone.0050067.

Taning, C.N.T., Andrade, E.C., Hunter, W.B., Christiaens, O., Smagghe, G. Asian citrus psyllid RNAi pathway – RNAi evidence. *Sci. Rep. Nature* 2016, 6, 38082. Doi:10.1038/srep38082

Vosburg, C., Reynolds, M., Noel, R., Shippy, T., Hosmani, P.S., et al. Characterization of Wnt signaling genes in *Diaphorina citri*, Asian citrus psyllid. *bioRxiv*_2020, [Pre-Print], 2020.09.21.306100. Accessed April 23, 2021. https://doi.org/10.1101/2020.09.21.306100.

Wang, X-W., Luan, J-B., Li, J-M., Bao, Y-Y., Zhang, C-X., Liu, S-S. *De novo* characterization of a whitefly transcriptome and analysis of its gene expression during development. *BMC Genomics* 2010, 11**,** 400 (2010). https://doi.org/10.1186/1471-2164-11-400

Wosula, E.N., Chen, W., Fei, Z., Legg, J.P. Unravelling the genetic diversity among cassava *Bemisia tabaci* whiteflies using NextRAD sequencing. *Genome Biol. and Evol*. 2017, 9(11), 2958–2973.

Xie, W., Chen, C., Yang, Z., Guo, L., Yang, X., et al. Genome sequencing of the sweetpotato whitefly *Bemisia tabaci* MED/Q. *GigaScience* 2017, 6(5), 1-7. Doi:10.1093/gigascience/gix018.

Xu, H. J., Chen, T., Ma, X. F., Xue, J., Pan, P. L., Zhang, X. C., et al. Genome-wide screening for components of small interfering RNA (siRNA) and micro-RNA (miRNA) pathways in the brown planthopper, *Nilaparvata lugens* (Hemiptera: Delphacidae). *Insect Mol. Biol.* 2013, 22, 635–647. Doi: 10.1111/imb.12051

Yang, C, Ou, D., Guo, W., Lü, J., Guo, C., et al. *De novo* assembly of the Asian citrus psyllid *Diaphorina citri* (Hemiptera: Psyllidae) transcriptome across developmental stages. *Int. J. Mol. Sci*. 2020, 21(14), 4974. Doi:10.3390/ijms21144974.
